# Measurement of cumulative high-sensitivity C-reactive protein and monocyte to high-density lipoprotein ratio in the risk prediction of type 2 diabetes: a prospective cohort study

**DOI:** 10.1186/s12967-024-04895-4

**Published:** 2024-01-28

**Authors:** Dan Wu, Genyuan Chen, Yulong Lan, Shuohua Chen, Xiong Ding, Chiju Wei, Lois Balmer, Wei Wang, Shouling Wu, Wencan Xu

**Affiliations:** 1 Department of Endocrinology, The First Affiliated Hospital of Shantou University Medical College, NO. 57, Changping Road, Jinping District, Shantou, 515041 Guangdong China; 2 Department of Pediatrics, Second Affiliated Hospital of Shantou University Medical College, Shantou, 515041 China; 3 Centre for Precision Health, Edith Cowan University, Perth, WA 6027 Australia; 4 Department of Endocrinology, Chaoan People’s Hospital, Longhua Road, Chaoan, Chaozhou, 515647 China; 5 Department of Cardiology, Second Affiliated Hospital of Shantou University Medical College, Shantou, 515041 China; 6 Department of Cardiology, Kailuan General Hospital, Xinghua East Road, Tangshan, 063000 Hebei China; 7 School of Public Health, Wuhan University, Wuhan, 430072 China; 8 Multidisciplinary Research Center, Shantou University, Shantou, 515041 China; 9 Beijing Key Laboratory of Clinical Epidemiology, School of Public Health, Capital Medical University, Beijing, 100069 China; 10 School of Public Health, Shandong First Medical University & Shandong Academy of Medical Sciences, Tai’an, Shandong China

**Keywords:** Type 2 diabetes, Monocyte-to-high density lipoprotein ratio, Inflammation, Biomarker, Longitudinal cohort study

## Abstract

**Background:**

Converging data have suggested that monocytic inflammation and C-reactive protein (CRP) are biologically intertwined processes and are involved in diabetogenesis. This study aimed to investigate the association between systemic inflammation assessed by joint cumulative high-sensitivity C-reactive protein (CumCRP) and monocyte to high-density lipoprotein ratio (CumMHR) and incident type 2 diabetes (T2D) and their predictive value for T2D in a general population.

**Methods:**

A total of 40,813 nondiabetic participants from a prospective real-life cohort (*Kailuan Study*, China) were followed biennially from 2010/2011 until December 31, 2020. Multivariable Cox regression analyses were conducted to evaluate the adjusted hazard ratios (aHRs) of incident diabetes.

**Results:**

During a median follow-up of 7.98 (IQR: 5.74–8.87) years, 4848 T2D cases developed. CumMHR and CumCRP were alone or jointly associated with incident T2D after adjusting for potential confounders. Elevated CumMHR levels significantly increased the risk of incident diabetes in each CumCRP strata (*P*-interaction: 0.0278). Participants with concomitant elevations in CumMHR and CumCRP levels had the highest risk (aHR: 1.71, 95% CI 1.52–1.91) compared to both in the low strata. Notably, the coexposure-associated T2D risk was modified by age, sex, hypertension, dyslipidemia, and prediabetes status. C-statistics increased from 0.7377 to 0.7417 when CumMHR and CumCRP were added into the multivariable-adjusted model, with a net reclassification improvement (%) of 12.39 (9.39–15.37) (*P* < 0.0001).

**Conclusions:**

Cumulative hsCRP and MHR were both independently and jointly associated with an increased risk of T2D and their addition to established risk factors should improve risk prediction and reclassification of diabetes.

**Supplementary Information:**

The online version contains supplementary material available at 10.1186/s12967-024-04895-4.

## Background

The high prevalence of type 2 diabetes has created a tremendous health burden worldwide [1]. The silent progressive and lifelong nature of diabetes emphasizes the need for epidemiological investigation to provide a framework for the identification and stratification of at-risk populations from the perspectives of predicative, preventive and personalized/precise interventions [2].

Over time, metabolic inflammation contributes to diabetogenesis and has become a common hallmark in overt hyperglycemic settings [3, 4]. Currently, the most commonly used inflammatory marker for predicting the risk of type 2 diabetes is high-sensitivity C-reactive protein (hsCRP)/CRP [5, 6]. Nevertheless, emerging epidemiological data have suggested that the joint assessment of biomarkers, rather than each in isolation, improves the predictive power of diabetes risk and diabetic complications [3, 7]. Genetic findings have also demonstrated that inflammation may play a causal role in metabolic diseases via its upstream effectors instead of through its downstream biomarker CRP [8]. In addition to being a marker of inflammation, CRP is an important regulator of inflammatory processes and is specifically engaged in monocyte-derived innate immunity [9]. On the one hand, the production of hsCRP is largely dependent on the response to monocytic cytokines, mainly interleukin (IL)-1β and its secondary cytokine IL-6 [10, 11]. On the other hand, CRP negatively mediates the release of monocytic cytokines and the generation of monocytes [12, 13]. Notwithstanding the well-established biological interplay between CRP and upstream factors of monocytic inflammation, limited studies have indicated that these factors are adjuncts to the risk of diabetes. The monocyte to high-density lipoprotein cholesterol (HDL-C) ratio (MHR), derived from routine blood and lipid tests, has emerged as a novel biomarker for metabolic inflammation [14], because of its potential to indicate deteriorations in proinflammatory status that are enhanced by an imbalance of monocytes and deficiency of HDL-C [14, 15]. In addition to being an independent predictor for incident cardiovascular disease (CVD) [16, 17], the MHR was established to be a potential tool candidate for predicting type 2 diabetesin our previous work [18]. Furthermore, the significant interaction between the cumulative MHR and hsCRP found in our previous study supports the potential for their combined usefor a more comprehensive inflammatory risk assessment.

Currently, little is known about the combination of hsCRP and MHR for the risk prediction of diabetes. To further contribute our work to this field, we therefore conducted an analysis based on a real-life, prospective cohort, the Kailuan Study to longitudinally assessed the independence, specificity, and magnitude of chronic inflammation [reflected by time-averaged cumulative hsCRP (CumCRP) and MHR (CumMHR) in 4 years] on the risk of developing type 2 diabetes among the general population in mainland China.

## Methods

### Study participants

The Kailuan Study is a large, ongoing, community-based, real-world, prospective cohort study in China, and its study protocol and procedures have been previously described in detail [19, 20]. Briefly, 101,510 participants aged 18–98 years were recruited to complete biennial health surveys beginning in 2006/2007; the latest health survey ended on December 31, 2020. All participants provided informed written consent. The current study was approved by the Kailuan General Hospital Ethics Committee, China (2006–05) and the Human Research Ethics Committee of Edith Cowan University (2021–03159-BALMER).

**Fig. 1:**
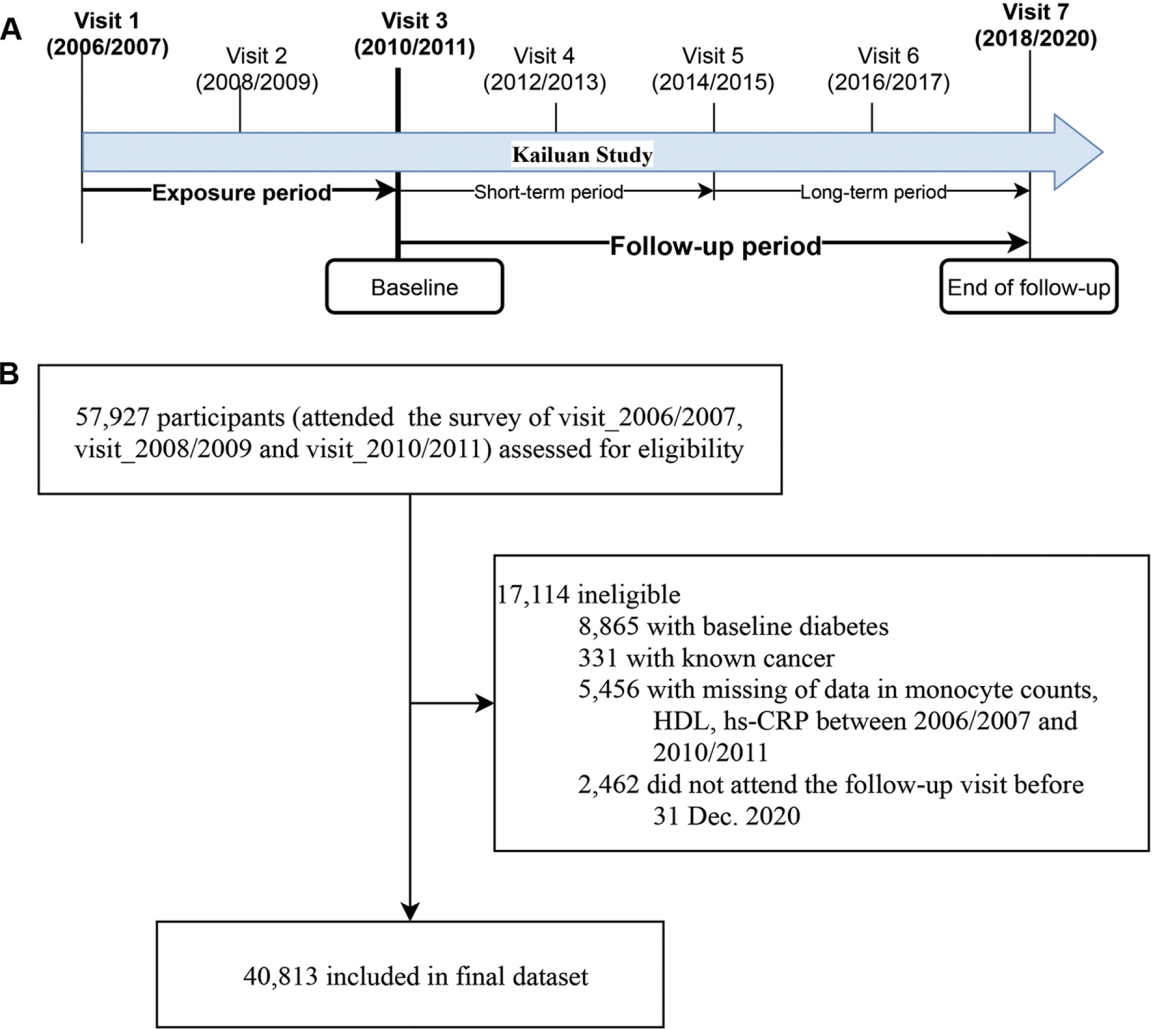
Study design and participant flow chart. **A** Design and strategy of the current study. **B** Flow chart of the study participants

The specific study design of the current analysis is presented in Fig. 1A, and a flowchart of the participants is shown in Fig. 1B. Among 57,927 original participants who attended the first three consecutive health surveys, 40,813 were recruited for this study. The exclusion criteria were as follows: participants with known diabetes (n = 8,865) or known cancer at baseline (n = 331); those with incomplete data or abnormal values in fasting blood glucose (FBG), monocyte count, hsCRP, and HDL-C during the exposure period (n = 5,456); and those who failed to contribute to their follow-up time from 2010/2011 through December 31, 2020 (n = 2,462). The numbers of participants who attended the follow-up visits and who underwent the glucose tests are provided in Additional file 1: Table S1.

### Assessment of the study outcome

The primary endpoint event of this study was the incidence of type 2 diabetes (International Classification of Diseases, 10th revision [ICD-10]: E11), defined as either FBG ≥ 7.0 mmol/L, a self-reported history of a physician diagnosis, or self-reported use of oral glucose-lowering medications with or without insulin use [21]. Participant death was documented by local government vital statistics offices. The date of diabetes onset was defined as the first follow-up examination at which a participant fulfilled the diagnostic criteria. The follow-up period lasted from the end of the baseline survey (2010/2011) until the date of diagnosis of type 2 diabetes, death, or the last available follow-up visit, whichever came first.

### Exposure

Chronic metabolic inflammation was assessed by measuring CumCRP and CumMHR during a median of 3.93 years of exposure [interquartile range (IQR): 3.73–4.26]. CumCRP was calculated as [(hsCRP1 + hsCRP2)/2 × (Visit2—Visit1)] + [(hsCRP2 + hsCRP3)/2 × (Visit3—Visit2)]/(Visit3-Visit1) [22–25], where hsCRP1, hsCRP2, and hsCRP3 correspond to hsCRP levels at each exposure visit. The CumMHR was calculated using the same algorithm, where the MHR = monocyte count/HDL-C. The time-averaged cumulative and mean values of the MHR and hsCRP are displayed in Additional file 1: Table S2. Given that there are there are no available clinical thresholds for CumCRP, the suggested clinical cutoff points for transient hsCRP (< 1, 1 to 3, and ≥ 3 mg/L connotes lower, average, and higher relative cardiometabolic risk, respectively [11]) were used for CumCRP analyses. Additionally, defining participants without and with elevated CumMHR was based on the 50th percentile (median) of the CumMHR values in the study population (0.2340), according to a previously established method [26]. To further evaluate the combined association between CumCRP and CumMHR and incident diabetes, the pooled sample was stratified according to CumCRP thresholds of < 1, 1 to 3 and ≥ 3 mg/L and CumMHR < 0.2340 or ≥ 0.2340 to create 6 joint exposure subgroups after confirmation of a significant interaction between CumMHR (< 0.2340 or ≥ 0.2340) and CumCRP (< 1, 1 to 3, or ≥ 3 mg/L) (*P* = 0.0278).

### Covariates

Potential covariates comprising sociodemographic and lifestyle characteristics as well as medical and medication history were collected via face-to-face interviews using a standard questionnaire, as described elsewhere [19, 20]. Anthropometrics, including height, weight, and blood pressure, were assessed by trained physicians. Laboratory assays on routine blood parameters (including leukocyte and monocyte counts), FBG, hsCRP level, total cholesterol (TC), HDL-C, low-density lipoprotein cholesterol (LDL-C), triglyceride (TG), and creatinine levels were conducted at the central laboratory of Kailuan General Hospital using a Hitachi 7600 autoanalyzer (Hitachi; Tokyo, Japan). The estimated glomerular filtration rate (eGFR) was calculated by the Chronic Kidney Disease Epidemiology Collaboration Creatinine Equation for assessing renal function [27]. The body mass index (BMI) was calculated as the weight (kg) divided by the height squared (m^2^), and participants were categorized as underweight, normal weight, overweight, or obese. Blood pressure was categorized as normal blood pressure, grade I hypertension, grade II hypertension, or grade III hypertension. Smoking status was divided into three categories: never, former, and current smokers. Alcohol consumption was categorized as yes (drinker) or no (nondrinker).

### Statistical analysis

All analyses were conducted using SAS version 9.4 (SAS Institute, Cary, NC, USA). For all analyses, two-tailed *P* values < 0.05 were considered statistically significant, with the exception of the interaction analysis, in which *P* < 0.1 was considered significant. Baseline characteristics are expressed as the mean ± standard deviation (SD), median and IQR, or number (percentage) for normally distributed, skewed and categorical data, respectively. These baseline characteristics are presented for the whole cohort and according to the CumMHR-by-CumCRP strata. Differences among subgroups were determined by one-way analyses of variance (ANOVAs), Kruskal–Wallis tests, or Pearson chi-square tests, as appropriate.

The incidence rates of type 2 diabetes were calculated by dividing the total number of events by the person-years of follow-up and were reported as events per 1000 person–years. Cox regression analyses were performed to determine the adjusted hazard ratios (aHRs) for type 2 diabetes and 95% confidence intervals (CIs), except for isolated CumCRP as the exposure (for which weighted Cox regression models were used because of the violation of the proportionality assumption). The multiplicative interaction between CumMHR and CumCRP were tested by a likelihood ratio test in the multivariable adjusted Cox regression model, which included both the main effects and the interaction term. To compare the cumulative incidence of type 2 diabetes across CumMHR-by-CumCRP strata, Kaplan–Meier plots were generated, and the log-rank test was conducted. The models were adjusted for the following variables: age, sex, smoking status, alcohol consumption, BMI, and family history of diabetes (Model 1); all the previous variables plus lipid-lowering medication and antihypertensive medication use, logTG, LDL-C, blood pressure, FBG, eGFR, and log(leukocyte count) (Model 2); Model 2 plus log(CumMHR) (Model 3); and Model 2 plus log(CumCRP) (Model 4). Further analyses were conducted to determine whether the strength of the association between coexposure and the risk of type 2 diabetes varied by age, sex, or other clinically relevant factors, including baseline dyslipidemia, hypertension, and impaired fasting glucose. Subgroup analyses were performed for variables that were significant for interaction. In addition, several sensitivity analyses were performed to assess the robustness of the findings. First, to address potential reverse causation, we excluded participants whose endpoints were reached and recorded at the first follow-up visit. Second, to minimize the influence of acute infection, participants with suspected acute infections (any participants with a hsCRP level ≥ 10 mg/L in the exposure period [11]) were excluded. Third, participants who took statins were excluded to prevent potential confounding effects of statin use from infusing the study endpoint. Fourth, to minimize the influence of CVD, participants with preexisting CVD were excluded.

Hereafter, the incremental value of CumCRP and CumMHR to improve risk prediction when they were added to classical diabetic risk factors was assessed by calculating Harrell’s C-statistic, and reclassification was assessed using the integrated discrimination improvement (IDI) and the continuous net reclassification improvement (NRI) [28].

## Results

Baseline characteristics were determined according to the information provided at the start of follow-up (Table 1). As anticipated, the mean age of the participants at the start of the follow-up period was 52.2 ± 11.8 years, and 30,634 (75.1%) were men. In terms of cumulative exposures, a higher CumMHR in each CumCRP strata was positively associated with higher cumulative FBG and hsCRP levels as well as monocyte counts. With regard to baseline characteristics, participants with higher CumMHR and CumCRP had high levels of BMI, hsCRP, TG, leukocyte counts, systolic and diastolic blood pressures, whereas had low levels of TC, HDL-C and eGFRs. Additionally, participants with higher CumMHR and higher CumCRP levels were more likely to be elderly, physical inactive, current smokers and current drinkers and have hypertension or dyslipidemia.

**Table 1:** Baseline characteristics of 40,813 participants

Characteristics	Total	CumMHR < 0.234 & CumCRP < 1 mg/L	CumMHR < 0.234 & 1 ≤ CumCRP < 3 mg/L	CumMHR < 0.234 & CumCRP ≥ 3 mg/L	CumMHR ≥ 0.234 & CumCRP < 1 mg/L	CumMHR ≥ 0.234 & 1 ≤ CumCRP < 3 mg/L	CumMHR ≥ 0.234 & CumCRP ≥ 3 mg/L	*P* value
Participants	40813	7743	8564	4099	5177	8848	6382	< 0.0001
Event-free time	8.0 (5.7–8.9)	8.2 (6.4–9.0)	7.9 (5.8–9.0)	8.0 (5.6–8.9)	8.0 (5.8–8.7)	7.9 (5.4–8.8)	7.8 (5.1–8.8)	< 0.0001
Cumulative characteristics								
CumMHR	0.2 (0.2–0.3)	0.2 (0.1–0.2)	0.2 (0.1–0.2)	0.2 (0.2–0.2)	0.3 (0.3–0.4)	0.3 (0.3–0.4)	0.3 (0.3–0.4)	< 0.0001
CumHDL, mmol/L	1.5 (1.3–1.8)	1.7 (1.4–1.9)	1.6 (1.4–1.9)	1.7 (1.4–1.9)	1.5 (1.3–1.6)	1.5 (1.3–1.6)	1.5 (1.3–1.7)	< 0.0001
CumMON, 10^9^/L	0.3 (0.3–0.4)	0.3 (0.2–0.3)	0.3 (0.2–0.3)	0.3 (0.2–0.3)	0.4 (0.4–0.5)	0.4 (0.4–0.5)	0.4 (0.4–0.5)	< 0.0001
CumCRP, mg/L	1.6 (0.8–3.1)	0.6 (0.4–0.8)	1.7 (1.3–2.2)	4.8 (3.7–6.7)	0.7 (0.5–0.8)	1.7 (1.3–2.2)	5.2 (3.9–7.7)	< 0.0001
Baseline characteristics								
Male, n (%)	30634 (75.1)	5138 (66.4)	5891 (68.8)	2660 (64.9)	4332 (83.7)	7482 (84.6)	5131 (80.4)	< 0.0001
Age, years	52.2 ± 11.8	51.2 ± 11.8	53.4 ± 11.8	56.7 ± 11.7	48.6 ± 10.5	50.6 ± 11.5	54.3 ± 12.0	< 0.0001
BMI, kg/m^2^	25.0 ± 3.3	23.7 ± 3.0	24.9 ± 3.2	25.3 ± 3.5	24.5 ± 3.0	25.5 ± 3.2	26.1 ± 3.6	< 0.0001
SBP, mmHg	129.6 ± 18.6	125.6 ± 18.2	130.1 ± 18.5	132.2 ± 19.4	127.0 ± 16.9	130.7 ± 18.3	133.0 ± 19.1	< 0.0001
DBP, mmHg	80.7 (79.3–90.0)	80.0 (74.0–90.0)	80.7 (80.0–90.0)	80.7 (80.0–90.0)	80.7 (79.0–90.0)	83.0 (80.0–90.0)	83.3 (80.0–90.0)	< 0.0001
FBG, mmol/L	5.2 ± 0.6	5.2 ± 0.6	5.3 ± 0.6	5.2 ± 0.6	5.2 ± 0.6	5.3 ± 0.6	5.2 ± 0.6	< 0.0001
HDL-C, mmol/L	1.5 (1.2–1.8)	1.7 (1.4–2.0)	1.7 (1.4–2.0)	1.6 (1.4–1.9)	1.4 (1.2–1.6)	1.4 (1.2–1.6)	1.3 (1.1–1.6)	< 0.0001
LDL-C, mmol/L	2.6 ± 0.8	2.6 ± 0.7	2.8 ± 0.7	2.3 ± 1.0	2.6 ± 0.7	2.7 ± 0.7	2.4 ± 0.9	< 0.0001
TC, mmol/L	5.0 ± 1.0	5.0 ± 1.0	5.1 ± 1.0	5.2 ± 1.0	4.8 ± 0.8	4.8 ± 0.9	4.9 ± 1.0	< 0.0001
TG, mmol/L	1.3 (0.9–1.8)	1.1 (0.8–1.5)	1.2 (0.9–1.8)	1.2 (0.9–1.8)	1.2 (0.9–1.8)	1.4 (1.0–2.1)	1.4 (1.0–2.2)	< 0.0001
HsCRP, mg/L	1.0 (0.5–2.4)	0.5 (0.3–0.9)	1.2 (0.7–2.1)	2.8 (1.0–5.8)	0.5 (0.1–0.9)	1.3 (0.6–2.4)	3.3 (1.3–6.6)	< 0.0001
Leukocytes, 10^9^/L	6.1 (5.2–7.2)	5.5 (4.7–6.4)	5.7 (4.8–6.6)	5.7 (4.8–6.7)	6.5 (5.6–7.5)	6.7 (5.8–7.9)	6.9 (5.9–8.0)	< 0.0001
Alcohol consumption, n (%)								< 0.0001
No	26752 (65.5)	5025 (64.9)	5624 (65.7)	3020 (73.7)	3110 (60.1)	5546 (62.7)	4427 (69.4)	
Yes	14061 (34.5)	2718 (35.1)	2940 (34.3)	1079 (26.3)	2067 (39.9)	3302 (37.3)	1955 (30.6)	
Smoking, n (%)								< 0.0001
Never smoker	25248 (61.9)	5160 (66.6)	5609 (65.5)	2913 (71.1)	2895 (55.9)	4881 (55.2)	3790 (59.4)	
Ever smoker	1796 (4.4)	320 (4.1)	355 (4.1)	176 (4.3)	223 (4.3)	422 (4.8)	300 (4.7)	
Current smoker	13769 (33.7)	2263 (29.2)	2600 (30.4)	1010 (24.6)	2059 (39.8)	3545 (40.1)	2292 (35.9)	
Family history of diabetes	2162 (5.3)	419 (5.4)	454 (5.3)	179 (4.4)	306 (5.9)	468 (5.3)	336 (5.3)	0.0481
Education, n (%)								< 0.0001
Less than high school	31,295 (76.7)	5504 (71.1)	6342 (74.1)	3256 (79.4)	3961 (76.5)	6953 (78.6)	5279 (82.7)	
High school and above	9518 (23.3)	2239 (28.9)	2222 (25.9)	843 (20.6)	1216 (23.5)	1895 (21.4)	1103 (17.3)	
Physical activities, n (%)								< 0.0001
Never	13749 (33.7)	2960 (38.2)	2990 (34.9)	1464 (35.7)	1705 (32.9)	2604 (29.5)	2026 (31.7)	
Occasionally	21,329 (52.3)	3547 (45.8)	4101 (47.9)	2061 (50.3)	2848 (55.0)	5134 (58.0)	3638 (57.0)	
Frequently	5735 (14.1)	1236 (16.0)	1473 (17.2)	574 (14.0)	624 (12.1)	1110 (12.5)	718 (11.3)	
Hypertension, n (%)	19733 (48.3)	2942 (38.0)	4111 (48.0)	2192 (53.5)	2226 (43.0)	4565 (51.6)	3705 (58.1)	< 0.0001
Dyslipidemia, n (%)	11100 (27.2)	1556 (20.1)	2186 (25.5)	1085 (26.5)	1317 (25.4)	2726 (30.8)	2224 (34.8)	< 0.0001
CVD, n (%)	2236 (5.48%)	297 (3.84%)	459 (5.36%)	291 (7.10%)	187 (3.61%)	506 (5.72%)	496 (7.77%)	< 0.0001
Antihypertensives, n (%)	2201 (5.4)	315 (4.1)	519 (6.1)	310 (7.6)	153 (3.0)	398 (4.5)	506 (7.9)	< 0.0001
Statin, n (%)	230 (0.6)	40 (0.5)	52 (0.6)	26 (0.6)	17 (0.3)	39 (0.4)	56 (0.9)	0.0014
Fibrate, n (%)	65 (0.2)	5 (0.1)	9 (0.1)	11 (0.3)	2 (0.0)	15 (0.2)	23 (0.4)	< 0.0001

*BMI* body mass index; *CumMHR* cumulative monocyte-to-high-density lipoprotein cholesterol ratio; *CumHDL* cumulative high-density lipoprotein cholesterol; *CumMON* cumulative monocytes; *DBP* diastolic blood pressure; *eGFR* estimated glomerular filtration rate; *FBG* fasting blood glucose; *HDL-C* high-density lipoprotein cholesterol; *hsCRP* high-sensitivity C-reactive protein; *SBP* systolic blood pressure; *TC* total cholesterol; *TG* triglyceride; *LDL-C* low-density lipoprotein cholesterol

The *P* value indicates intergroup comparison across the study subgroups

### Prospective analysis of type 2 diabetes incidence

During a median of 7.98 (IQR: 5.74–8.87) years of follow-up, 4,848 cases of type 2 diabetes occurred among the 40,813 study participants. In isolation, both the CumMHR and CumCRP were independently associated with the risk of type 2 diabetes. The aHR (95% CI) of incident diabetes per SD increase in logCumMHR (0.1995) in the fully adjusted model was 1.14 (1.10–1.18) in the entire population and differed across CumCRP strata: 1.13 (1.05–1.21), 1.10 (1.05–1.16) and 1.17 (1.10–1.24), in the CumCRP < 1, 1 ~ 3, ≥ 3 mg/L strata, respectively (Additional file 1: Table S3). With regard to CumCRP, a per-SD increase in logCumCRP (0.4295) conferred an aHR of 1.16 (95% CI: 1.13–1.19) for developing diabetes (Additional file 1: Table S4).

**Fig. 2:**
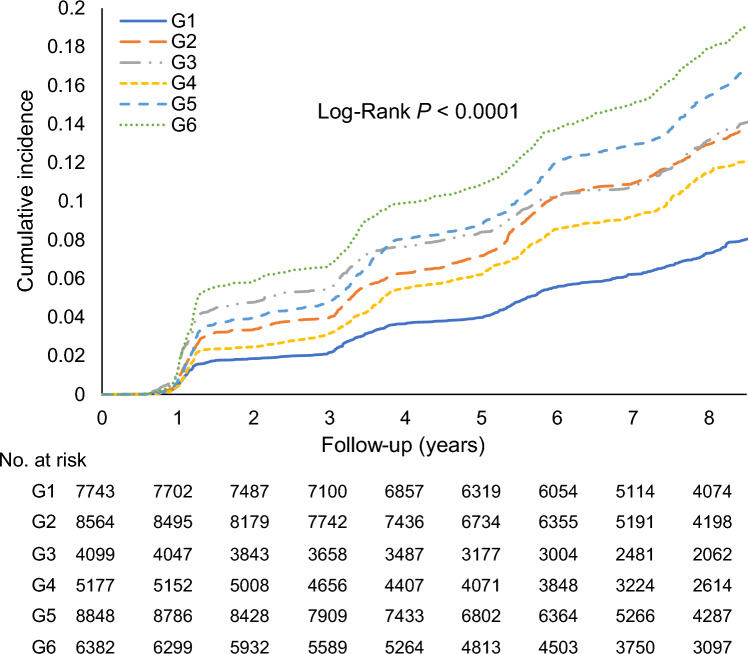
Cumulative incidence of type 2 diabetes across CumCRP-by-CumMHR strata**.** G1 CumCRP < 1 mg/L & CumMHR < 0.2340; G2 1 ≤ CumCRP < 3 mg/L & CumMHR < 0.2340; G3 CumCRP ≥ 3 mg/L & CumMHR < 0.2340; G4 CumCRP < 1 mg/L & CumMHR ≥ 0.2340; G5 1 ≤ CumCRP < 3 mg/L & CumMHR ≥ 0.2340; G6 CumCRP ≥ 3 mg/L & CumMHR ≥ 0.2340. Kaplan–Meier curves demonstrating the cumulative incidence and number at risk of diabetes across CumCRP-by-CumMHR subgroups in the entire population; No. at risk indicates the number of participants at specified time intervals with partially censored data

A significant interaction was detected between CumMHR and CumCRP; *P*-interaction: CumMHR (< 0.2340, or ≥ 0.2340) × CumCRP (< 1, 1 to 3, or ≥ 3 mg/L) = 0.0278; CumMHR (0.2340, or ≥ 0.2340) × log (CumCRP) < 0.0001 (Table 2). Concomitant elevations in both CumCRP and CumMHR levels significantly enhanced diabetic risk and incidence. Figure 2 displays the K–M curve comparing the cumulative incidence of type 2 diabetes. Compared to the reference group (CumCRP < 1 mg/L and CumMHR < 0.2340), participants with the same CumMHR level but with elevated CumCRP had significantly higher risks of type 2 diabetes, with aHRs (95% CIs) of 1.43 (1.29–1.59) and 1.42 (1.27–1.63) for 1 ≤ CumCRP < 3 and CumCRP ≥ 3 mg/L, respectively. Participants with normal CumCRP but elevated CumMHR (CumMHR ≥ 0.2340) had a significantly higher risk (1.32, 95% CI 1.16–1.49) than the reference group; those with elevated CumMHR and elevated CumCRP had aggressively higher risks of diabetes, with aHRs of 1.55 (1.39–1.72) and 1.71 (1.52–1.91) in the 1 ≤ CumCRP < 3 and CumCRP ≥ 3 mg/L strata, respectively. In each CumCRP stratum, an increased CumMHR significantly increased the risk of diabetes, while a decreased CumMHR reduced the risk (Table 2).

**Table 2:** The risk of incident type 2 diabetes upon exposure to cumulative MHR and cumulative hsCRP

	Combination of CumCRP and CumMHR, HRs (95% CIs)					
	CumCRP < 1 mg/L & CumMHR < 0.2340	1 ≤ CumCRP < 3 mg/L & CumMHR < 0.2340	CumCRP ≥ 3 mg/L & CumMHR < 0.2340	CumCRP < 1 mg/L & CumMHR ≥ 0.2340	1 ≤ CumCRP < 3 mg/L & CumMHR ≥ 0.2340	CumCRP ≥ 3 mg/L & CumMHR ≥ 0.2340
Event/Total	527/7743	1003/8564	500/4099	522/5177	1251/8848	1045/6382
Incidence rate	9.32	16.48	17.40	14.33	20.38	23.94
Unadjusted model	Reference	1.78 (1.60–1.98)	1.87 (1.66–2.12)	1.53 (1.35–1.73)	2.18 (1.97–2.42)	2.57 (2.31–2.85)
Model 1	Reference	1.53 (1.38–1.70)	1.48 (1.30–1.67)	1.43 (1.26–1.61)	1.78 (1.60–1.97)	1.88 (1.69–2.10)
Model 2	Reference	1.43 (1.29–1.59)	1.42 (1.27–1.63)	1.32 (1.16–1.49)	1.55 (1.39–1.72)	1.71 (1.52–1.91)
Model 3	Reference	1.26 (1.11–1.42)	1.10 (0.91–1.34)	1.35 (1.20–1.53)	1.41 (1.24–1.60)	1.35 (1.12–1.63)
Model 4	Reference	1.41 (1.27–1.57)	1.39 (1.23–1.58)	1.12 (0.97–1.30)	1.31 (1.15–1.48)	1.43 (1.25–1.64)
Model 1	0.65 (0.59–0.73)	Reference Reference	0.97 (0.87–1.08)	0.93 (0.84–1.04)	1.16 (1.07–1.27)	1.23 (1.13–1.34)
Model 2	0.70 (0.63–0.78)		1.01 (0.90–1.12)	0.93 (0.84–1.04)	1.11 (1.02–1.21)	1.22 (1.11–1.34)
Model 1	0.56 (0.51–0.62)	0.86 (0.79–0.94)	0.83 (0.75–0.92)	0.80 (0.72–0.89)	Reference	1.06 (0.97–1.15)
Model 2	0.65 (0.58–0.72)	0.92 (0.84–1.01)	0.92 (0.83–1.03)	0.85 (0.77–0.95)	Reference	1.10 (1.01–1.20)
Model 1	0.53 (0.48–0.59)	0.81 (0.74–0.89)	0.78 (0.70–0.87)	0.76 (0.68–0.84)	0.95 (0.87–1.02)	Reference
Model 2	0.58 (0.52–0.66)	0.84 (0.76–0.92)	0.84 (0.75–0.93)	0.77 (0.69–0.86)	0.91 (0.83–0.99)	Reference

*P*-interaction: CumMHR (< median or ≥ median) * CumCRP (< 1, 1 to 3, or ≥ 3 mg/L) = 0.0278; CumMHR (< median or ≥ median) * *log*(CumCRP) < 0.0001

Model 1: adjusted for age (continuous), sex, education, smoking status, drinking status, physical activity, family history of diabetes, and BMI (categorical);

Model 2: Model 1 + FBG (continuous), hypertension (categorical), *log*TG (continuous), LDL-C (continuous), eGFR (categorical), antihypertensives (yes or no), lipid-lowering drugs (yes or no), and *log*(leukocyte) (continuous);

Model 3: Model 2 + *log*CumCRP;

Model 4: Model 2 + *log*CumMHR. Abbreviations as Table 1

When follow-up was limited to approximately 4 years, the short-term risk of type 2 diabetes (HR [95% CI]) was 1.85 (1.58–2.16) in the CumCRP ≥ 3 mg/L with CumMHR ≥ 0.2340 subgroup, while the long-term risk (after excluding participants with diabetes onset within the first two follow-up visits) in this subgroup was 1.58 (1.34–1.86) (Additional file 1: Table S5).

**Fig. 3:**
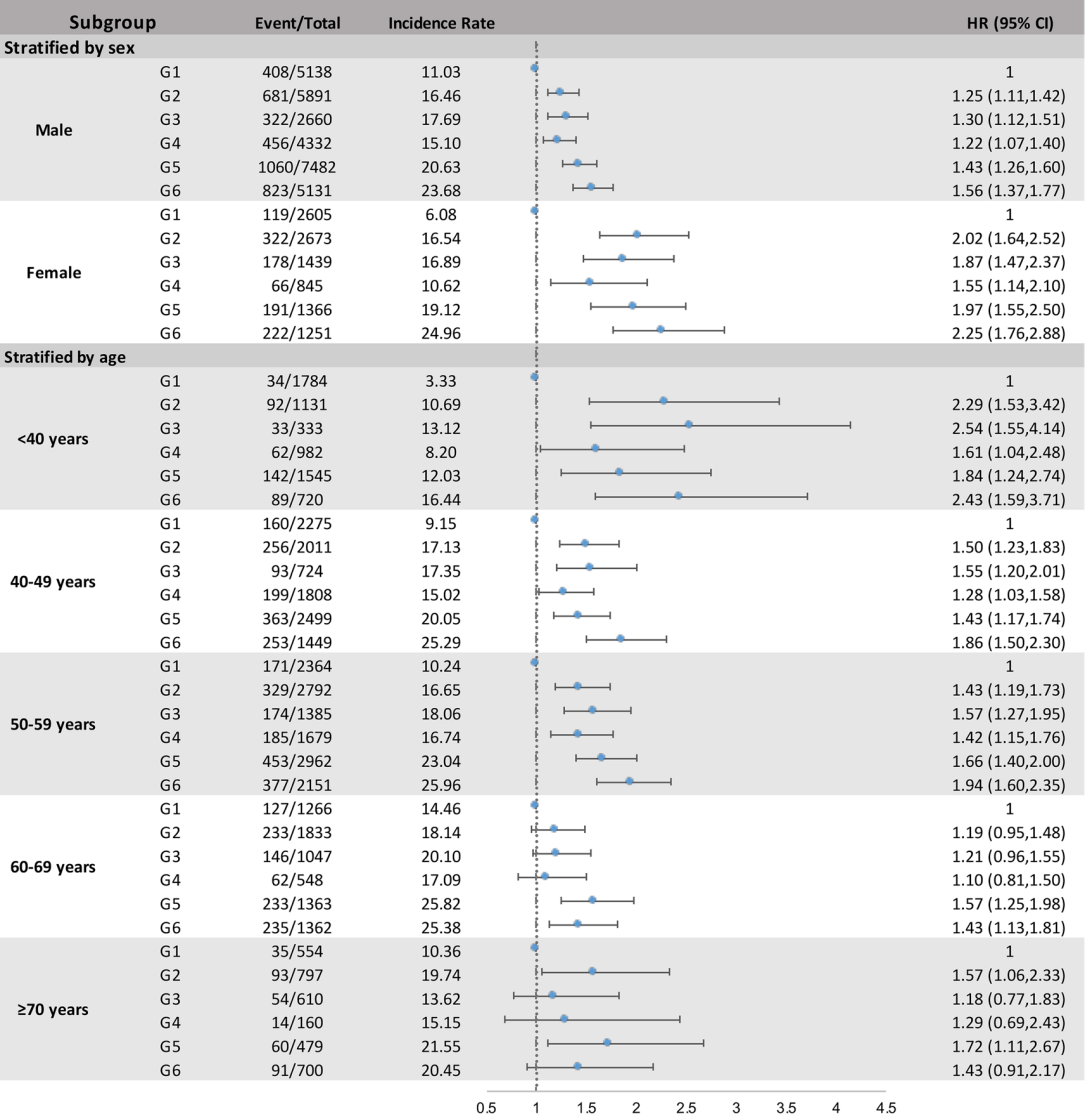
Forest plot of the risk of incident type 2 diabetes across CumCRP-by-CumMHR strata stratified by sex and age. G1 CumCRP < 1 mg/L & CumMHR < 0.2340 G2 1 ≤ CumCRP < 3 mg/L & CumMHR < 0.2340; G3 CumCRP ≥ 3 mg/L & CumMHR < 0.2340; G4 CumCRP < 1 mg/L & CumMHR ≥ 0.2340; G5 1 ≤ CumCRP < 3 mg/L & CumMHR ≥ 0.2340; G6 CumCRP ≥ 3 mg/L & CumMHR ≥ 0.2340. *P*-interanion: CumCRP-by-CumMHR strata × sex (male/female) = 0.0032; CumCRP-by-CumMHR strata × age subgroups (< 40, 40 ~ 49, 50 ~ 59, 60 ~ 69, or ≥ 70 years) = 0.0074. All models were adjusted for age (continuous), sex (except for sex subgroup analysis), education, smoking status, drinking status, physical activity, family history of diabetes, BMI (categorical), FBG (continuous), hypertension (categorical), *log*TG (continuous), LDL-C (continuous), eGFR (categorical), use of antihypertensives (yes or no), use of lipid-lowering drugs (yes or no), and *log*(leukocyte) (continuous). Abbreviations as in Table 1

In the present study, there was significant heterogeneity in the sex-associated risk of diabetes conferred by combined chronic inflammatory exposure; *P*-interaction: CumCRP-by-CumMHR strata × sex = 0.0032 (Fig. 3, Additional file 1: Table S6). The aHRs (95% CIs) were 1.30 (1.12–1.51) for men and 1.87 (1.47–2.37) for women with CumCRP ≥ 3 mg/L and a low CumMHR (< 0.2340) and were 1.56 (1.37–1.77) for males and 2.25 (1.76–2.88) for females with CumCRP ≥ 3 mg/L and a high CumMHR (≥ 0.2340). The interaction between the CumMHR and CumCRP in men (*P*-interaction = 0.6447) was not as significant as that in women (*P*-interaction = 0.0261). When examining the age-related heterogeneity in the risk of diabetes conferred by coexposure, we observed a positive increasing trend in diabetes incidence rates with increasing age, particularly in those aged 50–70 years. The highest CumCRP and CumMHR strata were jointly associated with the highest risk of diabetes across age subgroups. However, the risk of diabetes differed markedly by age subgroup; *P*-interaction: CumMHR-by-CumCRP strata × age subgroup (< 40, 40–49, 50–59, 60–69, ≥ 70 years) = 0.0074 (Fig. 3, Additional file 1: Table S7). Participants aged < 40 years had a markedly high risk (3.10, 95% CI: 2.06–4.68) after adjusting for sex, age, BMI, education, smoking, drinking habits and family history of diabetes. The risk was further attenuated in the fully adjusted model, with aHRs (95% CIs) of 2.43 (1.59–3.71), 1.86 (1.50–2.30), 1.94 (1.60–2.35), 1.43 (1.13–1.81) and 1.43 (0.91–2.17) for participants aged < 40, 40–49, 50–59, 60–69, and ≥ 70 years, respectively. Notably, the significant interaction between CumMHR and CumCRP persisted in those younger than 50 years but disappeared in those older than 50 years.

Participants’ dyslipidemia, hypertensive status or prediabetic status further modified the risk of incident diabetes upon chronic inflammatory exposure to CumMHR and CumCRP. The CumMHR × CumCRP interaction and the association between joint inflammatory exposure and incident diabetes were attenuated in participants with dyslipidemia, hypertension, or impaired fasting glucose in comparison to those without (Additional file 1: Tables S8-S10).

In the sensitivity analyses, the CumMHR × CumCRP interaction and the association between joint inflammatory exposure and incident diabetes remained significant when excluding participants with suspected infection, those treated with statins, those with preexisting CVD, or those with diabetes onset at the first follow-up visit (Additional file 1: Table S11).

Additionally, concomitant elevations in the baseline MHR and hsCRP levels were associated with increased risk and incidence of diabetes (Additional file 1: Table S12). The interaction between BasMHR (< median, or ≥ median) and BasCRP (< 1, 1 to 3, or ≥ 3 mg/L) was not statistically significant, whereas it was significant when BasCRP was tested as a continuous variable (logBasCRP).

### Clinical utility

Among all the study participants, the addition of CumCRP and CumMHR to the traditional risk model for diabetes increased the predicted value of incident diabetes from 0.7377 (95% CI 0.7302–0.7451) to 0.7417 (95% CI 0.7343–0.7491), with an NRI (%) of 12.39 (9.39–15.37) (*P* < 0.0001) and an IDI (%) of 0.16 (0.11–0.22) (Table 3). Additionally, the predictive value of the multivariable model was significantly greater (*P* < 0.01) in the CumCRP < 1 stratum (C-statistic = 0.7621, 95% CI 0.7473–0.7769) than in the other CumCRP strata (Additional file 1: Table S13).

**Table 3:** Prediction performance in the entire population for the full-adjusted model and with the addition of cumulative MHR and hsCRP

Models	C-statistics (95% CI)	SE	NRI, % (95% CI)	*P* value	IGI, % (95% CI)	*P* value
Multivariable model	0.7377 (0.7302–0.7451)	0.00381	–	–	–	–
Multivariable model + baseline MHR	0.7380 (0.7305–0.7453)	0.00381	4.14 (1.14–7.13)	0.0069	0.01 (0.00–0.02)	0.0056
Multivariable model + cumulative MHR	0.7397 (0.7322–0.7471)	0.00379	8.36 (5.36–11.35)	< 0.0001	0.11 (0.07–0.15)	0.0004
Multivariable model + cumulative hsCRP	0.7408 (0.7333–0.7482)	0.00377	12.02 (9.02–15.01)	< 0.0001	0.07 (0.03–0.12)	0.0017
Multivariable model + cumulative MHR + Cumulative hsCRP	0.7417 (0.7343–0.7491)	0.00376	12.39 (9.39–15.37)	< 0.0001	0.16 (0.11–0.22)	< 0.0001

The Multivariable model:was adjusted for age (continuous), sex, education, smoking status, drinking status, physical activity, family history of diabetes, BMI (categorical), FBG (continuous), hypertension (categorical), *log*TG (continuous), LDL-C (continuous), eGFR (categorical), antihypertensives (yes or no), lipid-lowering drugs (yes or no), *log*(leukocyte) (continuous), and *log*(hsCRP)

The baseline MHR, cumulative MHR and cumulative hsCRP were all log-transformed and added in the models

*IDI* integrated discrimination improvement; *NRI* net reclassification improvement; others as in Table 1

## Discussion

In this longitudinal analysis of 40,813 participants free of preexisting diabetes, we observed a significant association between cumulative exposure to either an elevated MHR or hsCRP levels in isolation and incident type 2 diabetes. A significant interaction between CumMHR and CumCRP was observed. Specifically, increases in the CumMHR in each CumCRP stratum increased the risk of type 2 diabetes; concomitant increases in CumMHR and CumCRP conferred significantly higher incidence rates and risks of diabetes. Furthermore, the association between chronic inflammation (reflected by the joint cumulative MHR and hsCRP exposure) and incident diabetes was highly age- and sex-specific and influenced by hypertension, dyslipidemia or prediabetes. The addition of the CumMHR and CumCRP to the clinical risk model significantly improved the prediction of incident diabetes.

Particularly relevant to the current findings, compelling studies in recent years have consistently documented the extensive involvement of monocytic immunity in diabetogenesis, which induces islet inflammation, beta-cell malfunction and insulin resistance [29, 30]. Additionally, it is essential to know that the inflammatory response is a complex signaling network involving diverse inflammatory factors. Converging evidence has suggested that the combined effects of these factors are likely to be more important than those of factors in isolation [3, 7]. Consistent with this theory, we observed a significant interaction between CumMHR and CumCRP and an increased risk of diabetes conferred by joint increases in CumMHR and CumCRP compared to that of each biomarker alone. The underlying mechanism of this association may be a bidirectional relationship between the monocytic inflammatory cascade and CRP levels. As a primary acute-phase reactant, CRP is produced largely dependent on the response to monocytic cytokines [11]. In turn, CRP mediates innate immunity [12] and monocyte generation [31]. Additionally, HDL-C metabolism significantly negatively influences monocytosis and attenuates monocytic inflammation [32]. Indeed, in the diabetes-prone milieu, a deficiency of HDL-C due to insulin resistance and lipid disorders is commonly observed [33, 34], suggesting the usefulness of the MHR as a biomarker for tracking an inflammatory imbalance preceding the occurrence of diabetes [18]. Taken together, our epidemiological observations, coupled with emerging experimental evidence, support the possibility that the biological interactions between monocytic inflammation and CRP may have functional consequences for diabetogenesis.

In the present study, the age-related attenuation in the diabetic risk conferred by coexposure to CumMHR and CumCRP was of interest. The occurrance of a significant CumCRP-CumMHR interaction was limited in participants aged < 50 years, and a markedly greater risk of type 2 diabetes (approximately 3 times greater) was observed in those aged < 40 years than in other age subgroups, indicating an age-dependent pattern of inflammation-associated risk of diabetes. Several interpretations may explain this finding. First, the downward trend in the risk of diabetes associated with joint exposure to CumCRP and CumMHR as age increases is consistent with the consensus that aging is the greatest risk factor for type 2 diabetes [2]. Type 2 diabetes is a typical age-related disease that mostly occurs in middle age and is partly attributed to the cumulative nature of age-associated inflammation, also known as “inflammaging” [35]. The increase in inflammation with increasing age may in part explain the decreasing trend in the inflammation-related risk of type 2 diabetes with aging. Our findings corroborate the involvement of chronic inflammation in the etiology of early-onset diabetes and merit specific attention. Epidemiological evidence indicates a consistent increase in early-onset diabetes, especially in developing countries [36]. Leveraging this age-specific association between chronic inflammation and type 2 diabetes may be a promising method for achieving early identification of at-risk young adults and developing personalized interventions.

In addition, we found significant sex differences the risk of diabetes conferred by coexposure to CumCRP and CumMHR. Compared with males, females had a greater risk of type 2 diabetes conferred by joint increases in CumCRP and CumMHR. Sex hormones may account for these sex differences [37, 38]. Previous studies have reported sex differences in the risk of diabetes associated with inflammatory markers, and these results support our findings [37, 38]. Our data reinforce the idea that monocyte-related inflammatory processes may be particularly important in diabetogenesis among women.

Moreover, the association between joint inflammatory exposure and incident diabetes was more pronounced in participants without hypertension, dyslipidemia or prediabetes. The attenuation of the inflammation-related risk for developing diabetes in these subsets of participants is likely because these factors greatly contribute to the occurrence of diabetes rather than inflammation per se. Taken together, our findings suggest that inflammatory exposure may be more important in the pathogenesis of diabetes among individuals in low-risk groups.

The surge in type 2 diabetes incidence in recent years has become a major health threat in the Chinese population [39] and is attributed to substantial changes in lifestyle, e.g., excess nutrient intake and increased sedentary behaviors, as a result of rapid economic development [40]. The chronic progressive nature of diabetes and the enormous burden of subsequent comorbidities further highlight the urgent need to address this critical public issue. Although aging and genetics are nonmodifiable risk factors, other risk factors can be modified through lifestyle changes [2]. The monocytic inflammation profile is strongly influenced by life activities and metabolic conditions, e.g., diet [41], sleep disruptions [42], chronic stress [43], and glucose and cholesterol dysregulation [30, 44], thereby indicating the potential benefits of monitoring risk-related metabolic conditions. Furthermore, the risk of type 2 diabetes conferred by concomitant increases in MHR and hsCRP levels was observed among low-risk participants, i.e., young, female, nonhypertensive, nondyslipidemic and nonprediabetic individuals, signaling that targeted assessment and management of joint cumulative MHR and hsCRP exposure may be especially important for further reducing risk of incident diabetes. Importantly, the significant improvement in predicting diabetes onset by the addition of cumulative MHR and hsCRP into traditional risk factors and the significantly high predicted value in the CumCRP < 1 stratum highlighted the need for ongoing evaluation of the inflammatory risks for a precise prediction of type 2 diabetes. The dual advantages of cost-effectiveness and wide availability of cumulative MHR and hsCRP in the current clinical setting potentiate their widespread use as convenient tools for risk prediction of diabetes.

The strengths of the current study include longitudinally examining the influence of metabolic inflammation over time on the development of diabetes. Although prolonged inflammatory exposure is the most important factor for cardiometabolic diseases, prior studies in this field have been mostly based on transient exposure or cross-sectional data, which may lead to the potential for underestimating the true association between chronic inflammation and type 2 diabetes. Additionally, controversies regarding the stability of hsCRP levels over time [10, 19] and the vulnerability of peripheral monocyte pools [20, 21] require repeated measurements to determine the stability and validity of the results. Another merit of this study is the assessment of systemic inflammation by the combination of monocytic inflammation and hsCRP levels. The inflammatory response is a complicated network involving multiple factors, suggesting that the use of other inflammatory biomarkers, in addition to the commonly used hsCRP levels, may allow a more comprehensive assessment of inflammation-related risks. Furthermore, the ability to extend the current understanding of this topic among specific age and sex subgroups is a unique contribution to the literature.

Limitations of the current study should be noted. First, this study primarily comprised participants from an occupation-based community among the Han Chinese population, which potentially limits the generalizability of the findings to the whole country or to other ethnicities/races. However, the relative homogeneity of the study population in terms of inflammatory exposure enhanced the internal validity of our findings. Second, we could not distinguish type 1 from type 2 diabetes, although misclassification is likely to be minimal, as type 2 diabetes is the predominant form of diabetes (> 95%), and the average age of the study participants was greater than the onset age of type 1 diabetes. Third, we did not investigate the confounding effects of oral hormone replacement therapy on the background levels of the MHR and hsCRP in postmenopausal women, which may have led to bias in the study outcome among these women. Fourth, data on hemoglobin A1c concentrations for the diagnosis of type 2 diabetes were lacking, potentially resulting in underestimation of the true incidence of type 2 diabetes. Fifth, to provide reliable results regarding the association between study exposures and incident diabetes, we included individuals with complete data on the study exposure and outcomes, which may have inevitably introduced selection bias. Sixth, the study examined inflammatory exposure during a given period before follow-up, without calculating the cumulative exposure from baseline to the occurrence of diabetes or the last available follow-up visit. Further studies in this topic are warranted to minimize within‐follow‐up variation and to evaluate longstanding inflammatory status. Seventh, although we excluded participants with statin use as a sensitivity analysis to address the potential confounding effect, it is notable that the use of statins reported in the study participants was strikingly low and may have led to some bias. The reasons for the low prevalence of statin use reported at baseline may include potential recall bias, region- and nation-specific differences in drug resources and policies, the low prevalence of existing cardiometabolic diseases among the study participants and patient compliance in that era. Evaluation of the reproducibility of the existing findings in other populations is needed.

## Conclusions

Our study is the first attempt to disentangle the epidemiological interaction between monocytic inflammation and hsCRP levels and to investigate the utility of their combined use for predicting type 2 diabetes. In light of our findings, ongoing monitoring of the MHR and hsCRP levels over time might provide a supplemental method for the early determination of the risk of type 2 diabetes, especially for individuals at low risk defined by traditional risk factors.

### Supplementary Information

**Additional file 1: Table S1.** Number of participants and participations in the follow-up visits.** Table S2.** Comparison of cumulative MHR, hsCRP to their mean value in the health visits in the exposure period.**Table S3.** Incidence of diabetes according to CumMHR quartiles in the entire cohort and stratifying by CumCRP strata (1, 3 mg/L).**Table S4.** Incidence of diabetes Cumcording to CumCRP strata5.**Table S5.** Long-term and Short-term diabetic risks of joint exposure to CumMHR and CumCRP.**Table S6.** Associations between joint exposure to CumMHR and CumCRP with type 2 diabetes stratified by sex.** Table S7.** Associations between joint exposure to CumMHR and CumCRP with type 2 diabetes stratified by age.**Table S8.** Associations between joint exposure to CumMHR and CumCRP with diabetes stratified by baseline dyslipidemia status.**Table S9.** Associations between joint exposure to CumMHR and CumCRP with diabetes stratified by hypertensive status in the exposure period.**Table S10.** Associations between joint exposure to CumMHR and CumCRP withdiabetes stratified by impaired fasting glucose status in the exposure period.**Table S11.** Sensitivity analysis of associations between joint exposure to CumMHR and CumCRP with type 2 diabetes.**Table S12.** Incidence of diabetes according to joint exposure to BasCRP and BasMHR.**Table S13.** C-statistics for incident diabetes predicted by the relevant risk factors and addition of CumMHR in each CumCRP stratum.**Fig. S1.** Flowchart of the study participants.**Fig. S2.** Design and strategy of the current study.**Fig. S3.** Cumulative incidence of type 2 diabetes across CumCRP-by-CumMHR strata

## Data Availability

The datasets used and/or analyzed during the current study are available from the corresponding author upon reasonable request.

## Abbreviations

aHRs: Adjusted hazard ratios
BMI: Body mass index
CIs: Confidence intervals
CumCRP: Cumulative high-sensitivity C-reactive protein
CumMHR: Cumulative monocyte-to-HDL-C ratio
CumMON: Cumulative monocyte count
CVDs: Cardiovascular diseases
eGFR: Estimated glomerular filtration rate
FBG: Fasting blood glucose
HDL-C: High-density lipoprotein cholesterol
hsCRP: High-sensitivity C-reactive protein
IL: Interleukin
LDL-C: Low-density lipoprotein cholesterol
MHR: Monocyte to high-density lipoprotein cholesterol ratio
IQR: Interquartile range
SD: Standard deviation
TC: Total cholesterol
TG: Triglyceride
