# Supplementary material for: Measurement of cumulative high-sensitivity C-reactive protein and monocyte to high-density lipoprotein ratio in the risk prediction of type 2 diabetes: a prospective cohort study
Source: J Transl Med. 2024 Jan 28;22:110. doi: 10.1186/s12967-024-04895-4 (PMC10822164; doi:10.1186/s12967-024-04895-4)
Supplement: Supplementary file 1 — Additional file 1: Table S1. Number of participants and participations in the follow-up visits. Table S2. Comparison of cumulative MHR, hsCRP to their mean value in the health visits in the exposure period.Table S3. Incidence of diabetes according to CumMHR quartiles in the entire cohort and stratifying by CumCRP strata (1, 3 mg/L).Table S4. Incidence of diabetes Cumcording to CumCRP strata5.Table S5. Long-term and Short-term diabetic risks of joint exposure to CumMHR and CumCRP.Table S6. Associations between joint exposure to CumMHR and CumCRP with type 2 diabetes stratified by sex. Table S7. Associations between joint exposure to CumMHR and CumCRP with type 2 diabetes stratified by age.Table S8. Associations between joint exposure to CumMHR and CumCRP with diabetes stratified by baseline dyslipidemia status.Table S9. Associations between joint exposure to CumMHR and CumCRP with diabetes stratified by hypertensive status in the exposure period.Table S10. Associations between joint exposure to CumMHR and CumCRP withdiabetes stratified by impaired fasting glucose status in the exposure period.Table S11. Sensitivity analysis of associations between joint exposure to CumMHR and CumCRP with type 2 diabetes.Table S12. Incidence of diabetes according to joint exposure to BasCRP and BasMHR.Table S13. C-statistics for incident diabetes predicted by the relevant risk factors and addition of CumMHR in each CumCRP stratum.Fig. S1. Flowchart of the study participants.Fig. S2. Design and strategy of the current study.Fig. S3. Cumulative incidence of type 2 diabetes across CumCRP-by-CumMHR strata [file 12967_2024_4895_MOESM1_ESM.docx]

**Additional Material**

Table S1. Number of participants and participations in the follow-up visits 2

Table S2. Comparison of cumulative MHR, hsCRP to their mean value in the health visits in the exposure period 3

[Table S3. Incidence of diabetes according to CumMHR quartiles in the entire cohort and stratifying by CumCRP strata (1, 3 mg/L) 4](#_Toc128477654)

[Table S4. Incidence of diabetes Cumcording to CumCRP strata 5](#_Toc128477655)

[Table S5. Long-term and Short-term diabetic risks of joint exposure to CumMHR and CumCRP 6](#_Toc128477656)

[Table S6. Associations between joint exposure to CumMHR and CumCRP with type 2 diabetes stratified by sex 7](#_Toc128477657)

[Table S7. Associations between joint exposure to CumMHR and CumCRP with type 2 diabetes stratified by age 8](#_Toc128477658)

[Table S8. Associations between joint exposure to CumMHR and CumCRP with diabetes stratified by baseline dyslipidemia status 10](#_Toc128477659)

[Table S9. Associations between joint exposure to CumMHR and CumCRP with diabetes stratified by hypertensive status in the exposure period 11](#_Toc128477660)

[Table S10. Associations between joint exposure to CumMHR and CumCRP withdiabetes stratified by impaired fasting glucose status in the exposure period 12](#_Toc128477661)

[Table S11. Sensitivity analysis of associations between joint exposure to CumMHR and CumCRP with type 2 diabetes 13](#_Toc128477662)

[Table S12. Incidence of diabetes according to joint exposure to BasCRP and BasMHR 15](#_Toc128477663)

[Table S13 C-statistics for incident diabetes predicted by the relevant risk factors and addition of CumMHR in each CumCRP stratum 16](#_Toc128477664)

[Fig. S1. Flowchart of the study participants 17](#_Toc128477665)

[Fig. S2. Design and strategy of the current study 17](#_Toc128477666)

[Fig. S3. Cumulative incidence of type 2 diabetes across CumCRP-by-CumMHR strata 18](#_Toc128477667)

# Table S1. Number of participants and participations in the follow-up visits

| **Participations** | **Participants (40,813)** | **Participations of the glucose tests** | **Participants (40,691)** |
| --- | --- | --- | --- |
| 1 | 5076 | 1 | 7343 |
| 2 | 8457 | 2 | 8128 |
| 3 | 12420 | 3 | 11261 |
| 4 | 14860 | 4 | 13698 |

# Table S2. Comparison of cumulative MHR and hsCRP to their mean value in the health visits in the exposure period

|  | **Mean (SD)** | **MAX** | **MIN** | **p25** | **p50** | **P75** |
| --- | --- | --- | --- | --- | --- | --- |
| CumMHR | 0.2593 (0.1300) | 2.2077 | 0.0387 | 0.1719 | 0.2340 | 0.3160 |
| mMHR | 0.2557 (0.1222) | 2.8438 | 0.0440 | 0.1720 | 0.2322 | 0.3107 |
| CumCRP | 2.7672 (4.04） | 132.47 | 0.009 | 0.83 | 1.5677 | 3.0729 |
| mCRP | 2.510 (3.46） | 89.28 | 0.01 | 0.76 | 1.42 | 2.83 |

Abbreviation: CumMHR: time-averaged cumulative monocyte-to-high-density lipoprotein cholesterol ratio; mMHR: mean value of monocyte-to-high-density lipoprotein cholesterol ratio in three exposure visits; CumCRP: time-averaged cumulative high-sensitivity C-reactive protein; mCRP: mean value of high-sensitivity C-reactive protein in three exposure visits

# Table S3. Incidence of diabetes according to CumMHR quartiles in the entire cohort and stratifying by CumCRP strata (1, 3 mg/L)

|  | **CumMHR, HRs (95% CIs)** | | | | **P for trend** | **Per SD** |
| --- | --- | --- | --- | --- | --- | --- |
|  | **Quartile 1** | **Quartile 2** | **Quartile 3** | **Quartile 4** |  |  |
| Entire cohort | 4848/40813 |  |  |  |  |  |
| Event/Total | 890/10203 | 1140/10203 | 1253/10203 | 1565/10204 |  |  |
| Incidence rate | 12.11 | 15.68 | 17.52 | 22.38 |  |  |
| Unadjusted model | Reference | 1.29 (1.19–1.41) | 1.44 (1.32–1.57) | 1.83 (1.69–1.99) | <0.0001 | 1.24 (1.20–1.27) |
| Model 1 | Reference | 1.20 (1.10–1.31) | 1.29(1.18–1.41) | 1.59 (1.46–1.74) | <0.0001 | 1.18 (1.14–1.21) |
| Model 2 | Reference | 1.17 (1.07–1.28) | 1.21 (1.10–1.32) | 1.45 (1.32–1.60) | <0.0001 | 1.14 (1.10–1.18) |
| CumCRP<1 mg/L | 1049/12920 |  |  |  |  |  |
| Event/Total | 259/4365 | 268/3378 | 256/2909 | 266/2268 |  |  |
| Incidence rate | 8.12 | 10.87 | 12.39 | 16.89 |  |  |
| Model 1 | Reference | 1.25 (1.05–1.49) | 1.37 (1.15–1.63) | 1.85 (1.54–2.21) | <0.0001 | 1.22 (1.14–1.30) |
| Model 2 | Reference | 1.23 (1.04–1.47) | 1.31 (1.09–1.57) | 1.64 (1.36–1–96) | <0.0001 | 1.16 (1.08–1.24) |
| Model 3 | Reference | 1.20 (1.01–1.44) | 1.26 (1.04–1.51) | 1.54 (1.27–1.87) | <0.0001 | 1.13 (1.05–1.21) |
| 1≤CumCRP<3 mg/L | 2254/17412 |  |  |  |  |  |
| Event/Total | 437/4146 | 566/4418 | 553/4388 | 698/4460 |  |  |
| Incidence rate | 14.73 | 18.15 | 17.98 | 22.79 |  |  |
| Model 1 | Reference | 1.20 (1.05–1.36) | 1.17 (1.03–1.33) | 1.47 (1.29–1.66) | <0.0001 | 1.14 (1.09–1.19) |
| Model 2 | Reference | 1.18 (1.04–1.34) | 1.12 (0.99–1.28) | 1.41 (1.24–1.61) | <0.0001 | 1.12 (1.07–1.17) |
| Model 3 | Reference | 1.16 (1.02–1.32) | 1.09 (0.95–1.24) | 1.35 (1.18–1.55) | <0.0001 | 1.10 (1.05–1.16) |
| CumCRP≥3 mg/L | 1545/10481 |  |  |  |  |  |
| Event/Total | 194/1692 | 306/2407 | 444/2906 | 601/3476 |  |  |
| Incidence rate | 16.31 | 18.16 | 22.10 | 25.51 |  |  |
| Model 1 | Reference | 1.03 (0.86–1.23) | 1.21 (1.02–1.44) | 1.36 (1.15–1.61) | <0.0001 | 1.15 (1.09–1.22) |
| Model 2 | Reference | 1.03 (0.86–1.24) | 1.22 (1.03–1.45) | 1.38 (1.16–1.63) | <0.0001 | 1.17 (1.10–1.23) |
| Model 3 | Reference | 1.02 (0.85–1.23) | 1.21 (1.01–1.44) | 1.35 (1.13–1.62) | <0.0001 | 1.17 (1.10–1.24) |

*P*-INTm: CumCRP (<1, 1 to 3, or ≥3 mg/L) *CumMHR quartile=0.0466; logCumCRP*CumMHR quartile<0.0001.

Model 1: adjusted for age (continuous), sex, education, smoking status, drinking status, physical activity, family history of diabetes, and BMI (categorical).

Model 2: Model 1+ FBG (continuous), hypertension (categorical), *log*TG (continuous), LDL-C (continuous), eGFR(categorical), antihypertensives (yes or no), lipid-lowering drugs (yes or no), log(leukocyte) (continuous), *log*hsCRP(continuous) (limited to in the entire cohort).

Per SD: the diabetic risk per 1-SD increase in log(CumMHR)(0.1995);

Incidence rate is per 1,000 person-years.

Abbreviation: CumMHR: cumulative monocyte to high-density lipoprotein cholesterol ratio; BMI: body mass index; FBG: fasting blood glucose; eGFR: estimated glomerular filtration rate; TG: triglyceride; LDL-C: low-density lipoprotein cholesterol; hsCRP: high-sensitivity C-reactive protein.

# Table S4. Incidence of diabetes according to CumCRP strata

|  | **CumCRP, HRs (95% CIs)** | | | | ***P*-trend** | **Per SD** |
| --- | --- | --- | --- | --- | --- | --- |
|  | **CumCRP<1 mg/L** | **1≤CumCRP<3 mg/L** | **3≤CumCRP<10 mg/L** | **CumCRP≥10 mg/L** |  |  |
| Event/Total | 1049/12920 | 2254/17412 | 1332/8898 | 213/1583 |  |  |
| Incidence rate | 11.28 | 18.44 | 21.76 | 19.08 |  |  |
| Unadjusted model | Reference | 1.64 (1.53–1.77) | 1.93 (1.78–2.09) | 1.73 (1.49–2.00) | <0.0001 | 1.26 (1.23–1.30) |
| Model 1 | Reference | 1.38 (1.28–1.48) | 1.43 (1.32–1.55) | 1.29 (1.12–1.49) | <0.0001 | 1.14 (1.11–1.18) |
| Model 2 | Reference | 1.31 (1.22–1.41) | 1.42 (1.30–1.54) | 1.37 (1.18–1.59) | <0.0001 | 1.16 (1.13–1.19) |

Model 1: adjusted for age, sex, education, smoking status, drinking status, physical activity, family history of diabetes, and BMI (categorical).

Model 2: Model 1+FBG (continuous), eGFR (categorical), *log*(leukocytes) (continuous), *log*TG (continuous), LDL-C(continuous), blood pressure (categorical), antihypertensives (yes or no), and lipid-lowering drugs (yes or no).

Per SD: type 2 diabetes risk per SD increment in log(CumCRP) (0.4295);

Incidence rate is per 1,000 person-years.

Abbreviation: CumMHR: cumulative monocyte to high-density lipoprotein cholesterol ratio; CumCRP: cumulative high-sensitivity C-reactive protein; BMI: body mass index; FBG: fasting blood glucose; eGFR: estimated glomerular filtration rate; TG: triglyceride; LDL-C: low-density lipoprotein cholesterol.

# Table S5. Long-term and short-term risk of incident type 2 diabetes upon joint exposure to CumMHR and CumCRP

|  | **Combination of CumCRP and CumMHR，HRs (95% CIs)** | | | | | |
| --- | --- | --- | --- | --- | --- | --- |
|  | **CumCRP<1 mg/L & CumMHR<0.2340** | **1≤CumCRP<3 mg/L & CumMHR<0.2340** | **CumCRP≥3 mg/L & CumMHR<0.2340** | **CumCRP<1 mg/L & CumMHR≥0.2340** | **1≤CumCRP<3 mg/L & CumMHR≥0.2340** | **CumCRP≥3 mg/L & CumMHR≥0.2340** |
| **Short-term risk** | 2618/39584 |  |  |  |  |  |
| Event/Total | 265/7528 | 510/8311 | 301/3931 | 266/5046 | 677/8661 | 599/6157 |
| Incidence rate | 9.13 | 16.05 | 21.03 | 13.70 | 20.46 | 26.68 |
| Unadjusted model | Reference | 1.77 (1.52–2.05) | 2.30 (1.95–2.71) | 1.53(1.29–1.81) | 2.27(1.97–2.62) | 2.92 (2.53–3.38) |
| Model 1 | Reference | 1.48 (1.28–1.72) | 1.73 (1.46–2.05) | 1.44(1.21–1.70) | 1.83 (1.58–2.11) | 2.05 (1.77–2.38) |
| Model 2 | Reference | 1.40 (1.20–1.62) | 1.67 (1.41–1.97) | 1.33 (1.12–1.58) | 1.61 (1.38–1.86) | 1.85 (1.58–2.16) |
| *P*-interaction: CumMHR (< median, or ≥ median) ×CumCRP (<1, 1 to 3, or ≥3 mg/L) = 0.2508; CumMHR (< median, or ≥ median)×LogCumCRP<0.0001 | | | | | | |
| **Long-term risk** | 2230/38195 |  |  |  |  |  |
| Event/Total | 262/7478 | 493/8054 | 199/3798 | 256/4911 | 574/8171 | 446/5783 |
| Incidence rate | 4.68 | 8.24 | 7.05 | 7.14 | 9.58 | 10.49 |
| Unadjusted model | Reference | 1.80 (1.55–2.09) | 1.53 (1.27–1.84) | 1.54 (1.30–1.83) | 2.09 (1.81–2.42) | 2.28 (1.96–2.66) |
| Model 1 | Reference | 1.59 (1.36–1.85) | 1.27 (1.06–1.53) | 1.44 (1.21–1.71) | 1.74 (1.49–2.02) | 1.75 (1.50–2.05) |
| Model 2 | Reference | 1.47 (1.27–1.71) | 1.21 (1.01–1.47) | 1.32 (1.11–1.58) | 1.50 (1.28–1.74) | 1.58 (1.34–1.86) |
| *P*-interaction: CumMHR (< median, or ≥ median) ×CumCRP (<1, 1 to 3, or ≥3 mg/L) = 0.0190; CumMHR (< median, or ≥ median) ×LogCumCRP=0.0011 | | | | | | |

Model 1: adjusted for age (continuous), sex, education, smoking status, drinking status, physical activity, family history of diabetes, and BMI (categorical);

Model 2: Model 1+ FBG (continuous), hypertension (categorical), logTG (continuous), LDL-C(continuous), eGFR (categorical), antihypertensives (yes or no), lipid-lowering drugs (yes or no), and log(leukocyte) (continuous).

Abbreviation: CumMHR: cumulative monocyte to high-density lipoprotein cholesterol ratio; CumCRP: cumulative high-sensitivity C-reactive protein; BMI: body mass index; FBG: fasting blood glucose; eGFR: estimated glomerular filtration rate; HC: hipline circumference; WC: waist circumference; TG: triglyceride; LDL-C: low-density lipoprotein cholesterol.

# Table S6. Associations between joint exposure to CumMHR and CumCRP and type 2 diabetes stratified by sex

|  | **Combination of CumCRP and CumMHR，HRs (95% CIs)** | | | | | | ***P-*interaction** |
| --- | --- | --- | --- | --- | --- | --- | --- |
|  | **CumCRP<1 mg/L & CumMHR<0.2340** | **1≤CumCRP<3 mg/L & CumMHR<0.2340** | **CumCRP≥3 mg/L & CumMHR<0.2340** | **CumCRP<1 mg/L & CumMHR≥0.2340** | **1≤CumCRP<3 mg/L & CumMHR≥0.2340** | **CumCRP≥3 mg/L & CumMHR≥0.2340** |  |
| **Male** | 3750/30634 |  |  |  |  |  | 0.0032 |
| Event/Total | 408/5138 | 681/5891 | 322/2660 | 456/4332 | 1060/7482 | 823/5131 |  |
| Incidence rate | 11.03 | 16.46 | 17.69 | 15.10 | 20.63 | 23.68 |  |
| Unadjusted model | Reference | 1.50 (1.32–1.69) | 1.60 (1.39–1.86) | 1.36 (1.19–1.55) | 1.86 (1.66–2.09) | 2.14 (1.90–2.41) |  |
| Model 1 | Reference | 1.33 (1.17–1.50) | 1.34 (1.15–1.55) | 1.31 (1.4–1.49) | 1.60 (1.42–1.79) | 1.67 (1.48–1.88) |  |
| Model 2 | Reference | 1.25 (1.11–1.42) | 1.30 (1.12– 1.51) | 1.22 (1.07–1.40) | 1.43 (1.26–1.60) | 1.56 (1.37–1.77) |  |
| *P*-interaction: CumMHR (< median, or ≥ median) ×CumCRP (<1, 1 to 3, or ≥3 mg/L)= 0.6447; CumMHR (< median, or ≥ median) ×LogCumCRP<0.0001 | | | | | | |  |
| **Female** | 1098/10179 |  |  |  |  |  |  |
| Event/Total | 119/2605 | 322/2673 | 178/1439 | 66/845 | 191/1366 | 222/1251 |  |
| Incidence rate | 6.08 | 16.54 | 16.89 | 10.62 | 19.12 | 24.96 |  |
| Unadjusted mode1 | Reference | 2.75 (2.23–3.39) | 2.80 (2.22–3.53) | 1.74 (1.29–2.35) | 3.16 (2.52–3.98) | 4.12 (3.30–5.15) |  |
| Model 1 | Reference | 2.19 (1.77–2.71) | 1.92 (1.51–2.43) | 1.67 (1.23–2.25) | 2.40 (1.90– 3.04) | 2.68 (2.13–3.38) |  |
| Model 2 | Reference | 2.02 (1.64–2.52) | 1.87 (1.47–2.37) | 1.55 (1.14–2.10) | 1.97 (1.55–2.50) | 2.25 (1.76–2.88) |  |
| *P*-interaction: CumMHR (< median, or ≥ median) ×CumCRP (<1, 1 to 3, or ≥3 mg/L) = 0.0261; CumMHR (< median, or ≥ median) ×LogCumCRP<0.0001 | | | | | | |  |

Model 1: adjusted for age (continuous), education, smoking status, drinking status, physical activity, family history of diabetes, and BMI (categorical);

Model 2: Model 1+ FBG (continuous), hypertension (categorical), logTG (continuous), LDL-C(continuous), eGFR(categorical), antihypertensives (yes or no), lipid-lowering drugs (yes or no), and log(leukocyte)(continuous).

Incidence rate is per 1,000 person-years.

Abbreviation: CumMHR: cumulative monocyte to high-density lipoprotein cholesterol ratio; CumCRP: cumulative high-sensitivity C-reactive protein; BMI: body mass index; FBG: fasting blood glucose; eGFR: estimated glomerular filtration rate; TG: triglyceride; LDL-C: low-density lipoprotein cholesterol.

# Table S7. Associations between joint exposure to CumMHR and CumCRP and type 2 diabetes stratified by age

|  | **Combination of CumCRP and CumMHR, HRs (95% CIs)** | | | | | | ***P-*interaction** |
| --- | --- | --- | --- | --- | --- | --- | --- |
|  | **CumCRP<1 mg/L & CumMHR<0.2340** | **1≤CumCRP<3 mg/L & CumMHR<0.2340** | **CumCRP≥3 mg/L & CumMHR<0.2340** | **CumCRP<1 mg/L & CumMHR≥0.2340** | **1≤ CumCRP <3 mg/L & CumMHR≥0.2340** | **CumCRP≥3 mg/L & CumMHR≥0.2340** |  |
| **<40 years** | 452/5995 |  |  |  |  |  | 0.0074 |
| Event/Total | 34/1784 | 92/1131 | 33/333 | 62/982 | 142/1545 | 89/720 |  |
| Incidence rate | 3.33 | 10.69 | 13.12 | 8.20 | 12.03 | 16.44 |  |
| Unadjusted model | Reference | 3.23 (2.18–4.78） | 3.97 (2.46–6.41) | 2.44 (1.61–3.71) | 3.59 (2.47–5.21) | 4.96 (3.34–7.36) |  |
| Model 1 | Reference | 2.55 (1.71–3.79) | 2.98 (1.84–4.84) | 2.01 (1.31–3.06) | 2.49 (1.69–3.67) | 3.10 (2.06–4.68) |  |
| Model 2 | Reference | 2.29 (1.53–3.42) | 2.54 (1.55–4.14) | 1.61 (1.04–2.48) | 1.84 (1.24–2.74) | 2.43 (1.59–3.71) |  |
| *P*-interaction: CumMHR (< median, or ≥ median) ×CumCRP (<1, 1 to 3, or ≥3 mg/L) = 0.0243; CumMHR (< median, or ≥ median) ×LogCumCRP<0.0001 | | | | | | |  |
| **40-49 years** | 1324/10766 |  |  |  |  |  |  |
| Event/Total | 160/2275 | 256/2011 | 93/724 | 199/1808 | 363/2499 | 253/1449 |  |
| Incidence rate | 9.15 | 17.13 | 17.35 | 15.02 | 20.05 | 25.29 |  |
| Unadjusted model | Reference | 1.89 (1.51–2.29) | 1.92 (1.49–2.48) | 1.63 (1.33–2.01) | 2.19 (1.97–2.67) | 2.79 (2.29–3.41) |  |
| Model 1 | Reference | 1.63 (1.33–1.98) | 1.58 (1.22–2.04) | 1.44 (1.16–1.77) | 1.70 (1.40–2.05) | 2.01 (1.64–2.46) |  |
| Model 2 | Reference | 1.50 (1.23–1.83) | 1.55 (1.20–2.01) | 1.28 (1.03–1.58) | 1.43 (1.17–1.74) | 1.86 (1.50–2.30) |  |
| *P*-interaction: CumMHR (< median, or ≥ median) ×CumCRP (<1, 1 to 3, or ≥3 mg/L) = 0.0688; CumMHR (< median, or ≥ median) ×LogCumCRP<0.0001 | | | | | | |  |
| **50-59 years** | 1689/13333 |  |  |  |  |  |  |
| Event/Total | 171/2364 | 329/2792 | 174/1385 | 185/1679 | 453/2962 | 377/2151 |  |
| Incidence rate | 10.24 | 16.65 | 18.06 | 16.74 | 23.04 | 25.96 |  |
| Unadjusted model | Reference | 1.64 (1.36–1.97) | 1.76 (1.43–2.18) | 1.63 (1.32–2.00) | 2.24 (1.88–2.67) | 2.53 (2.11–3.03) |  |
| Model 1 | Reference | 1.50 (1.24–1.80) | 1.58 (1.28–1.96) | 1.47 (1.19–1.81） | 1.86 (1.56–2.23) | 2.04 (1.70–2.46) |  |
| Model 2 | Reference | 1.43 (1.19–1.73) | 1.57 (1.27–1.95) | 1.42 (1.15–1.76) | 1.66 (1.40–2.00) | 1.94 (1.60–2.35) |  |
| *P*-interaction: CumMHR (< median, or ≥ median) ×CumCRP (<1, 1 to 3, or ≥3 mg/L) = 0.3030; CumMHR (< median, or ≥ median) ×LogCumCRP<0.0001 | | | | | | |  |
| **60-69 years** | 1036/7419 |  |  |  |  |  |  |
| Event/Total | 127/1266 | 233/1833 | 146/1047 | 62/548 | 233/1363 | 235/1362 |  |
| Incidence rate | 14.46 | 18.14 | 20.10 | 17.09 | 25.82 | 25.38 |  |
| Unadjusted model | Reference | 1.27 (1.02–1.58) | 1.38 (1.09–1.76) | 1.18 (0.87–1.60) | 1.80 (1.45–2.23) | 1.75 (1.41–2.17) |  |
| Model 1 | Reference | 1.17 (0.94–1.45) | 1.25 (0.98–1.59) | 1.12 (0.83–1.53) | 1.59 (1.28–1.99) | 1.51 (1.21–1.88) |  |
| Model 2 | Reference | 1.19 (0.95–1.48) | 1.21 (0.96–1.55) | 1.10 (0.81–1.50) | 1.57 (1.25–1.98) | 1.43 (1.13–1.81) |  |
| *P*-interaction: CumMHR (< median, or ≥ median) ×CumCRP (<1, 1 to 3, or ≥3 mg/L) = 0.4927; CumMHR (< median, or ≥ median) ×LogCumCRP=0.0571 | | | | | | |  |
| **≥70 years** | 347/3300 |  |  |  |  |  |  |
| Event/Total | 35/554 | 93/797 | 54/610 | 14/160 | 60/479 | 91/700 |  |
| Incidence rate | 10.36 | 19.74 | 13.62 | 15.15 | 21.55 | 20.45 |  |
| Unadjusted model | Reference | 1.91 (1.29–2.81) | 1.34 (0.88–2.06) | 1.44 (0.78–2.68) | 2.07 (1.38–3.14) | 2.01 (1.36–2.97） |  |
| Model 1 | Reference | 1.70 (1.15–2.51) | 1.19 (0.77–1.82) | 1.25 (0.07–2.33) | 1.77 (1.16–2.71) | 1.62 (1.08–2.42) |  |
| Model 2 | Reference | 1.57(1.06– 2.33) | 1.18 (0.77–1.83) | 1.29 (0.69–2.43) | 1.72 (1.11–2.67) | 1.43 (0.91–2.17) |  |
| *P*-interaction: CumMHR (< median, or ≥ median) ×CumCRP (<1, 1 to 3, or ≥3 mg/L) = 0.8881; CumMHR (< median, or ≥ median) ×LogCumCRP=0.7438 | | | | | | |  |

Model 1: adjusted for age (continuous), sex, education, smoking status, drinking status, physical activity, family history of diabetes, and BMI (categorical);

Model 2: Model 1+ hypertension (categorical), logTG (continuous), LDL-C(continuous), eGFR(categorical), antihypertensives (yes or no), lipid-lowering drugs (yes or no), and log(leukocyte)(continuous).

Incidence rate is per 1,000 person-years.

Abbreviation: CumMHR: cumulative monocyte to high-density lipoprotein cholesterol ratio; CumCRP: cumulative high-sensitivity C-reactive protein; BMI: body mass index; FBG: fasting blood glucose; eGFR: estimated glomerular filtration rate; TG: triglyceride; LDL-C: low-density lipoprotein cholesterol.

# Table S8. Associations between joint exposure to CumMHR and CumCRP and incident diabetes stratified by baseline dyslipidemia status

|  | **Combination of CumCRP and CumMHR，HRs (95% CIs)** | | | | | | ***P*-interaction** |
| --- | --- | --- | --- | --- | --- | --- | --- |
|  | **CumCRP<1 mg/L & CumMHR<0.2340** | **1≤CumCRP<3 mg/L & CumMHR<0.2340** | **CumCRP≥3 mg/L & CumMHR<0.2340** | **CumCRP<1 mg/L & CumMHR≥0.2340** | **1≤ CumCRP<3 mg/L & CumMHR≥0.2340** | **CumCRP≥3 mg/L & CumMHR≥0.2340** |  |
| **Dyslipidemia** | 1736/11100 |  |  |  |  |  | 0.0158 |
| Event/Total | 157/1554 | 329/2183 | 174/1088 | 182/1318 | 474/2731 | 420/2226 |  |
| Incident rate | 14.30 | 21.83 | 23.65 | 20.11 | 25.40 | 27.81 |  |
| Unadjusted model | Reference | 1.53 (1.27–1.85) | 1.66 (1.33–2.05) | 1.40 (1.13–1.74) | 1.77 (1.48–2.12) | 1.94 (1.62–2.33) |  |
| Model 1 | Reference | 1.38 (1.14–1.67) | 1.37 (1.10–1.70) | 1.34 (1.08–1.67) | 1.53 (1.27–1.84) | 1.54 (1.27–1.85) |  |
| Model 2 | Reference | 1.25 (1.04–1.52) | 1.29 (1.04–1.60) | 1.26 (1.01–1.57) | 1.37 (1.13–1.65) | 1.42(1.17–1.72) |  |
| *P*-interaction: CumMHR (< median, or ≥ median) ×CumCRP (<1, 1 to 3, or ≥3 mg/L) = 0.5131; CumMHR (< median, or ≥ median) ×LogCumCRP=0.0196 | | | | | | |  |
| **Nondyslipidemia** | 3112/29713 |  |  |  |  |  |  |
| Event/Total | 370/6189 | 674/6381 | 326/3011 | 340/3859 | 777/6117 | 625/4156 |  |
| Incident rate | 8.12 | 14.72 | 15.24 | 12.42 | 18.19 | 21.90 |  |
| Unadjusted model | Reference | 1.83 (1.61–2.07) | 1.88 (1.62–2.19) | 1.52(1.31–1.76） | 2.23 (1.97–2.53) | 2.69 (2.37–3.06) |  |
| Model 1 | Reference | 1.56 (1.37–1.77) | 1.48 (1.27–1.72) | 1.42 (1.22–1.64) | 1.83 (1.61–2.07) | 1.97 (1.73–2.25) |  |
| Model 2 | Reference | 1.55 (1.36–1.76) | 1.51 (1.30–1.76) | 1.37 (1.18–1.60) | 1.72 (1.51–1.95) | 2.00 (1.74–2.30) |  |
| *P*-interaction: CumMHR (< median, or ≥ median) ×CumCRP (<1, 1 to 3, or ≥3 mg/L) = 0.0293; CumMHR (< median, or ≥ median) ×LogCumCRP<0.0001 | | | | | | |  |

Model 1: adjusted for age (continuous), sex, education, smoking status, drinking status, physical activity, family history of diabetes, and BMI (categorical);

Model 2: Model 1+ FBG (continuous), hypertension (categorical), eGFR(categorical), antihypertensives (yes or no), lipid-lowering drugs (yes or no), and log(leukocyte)(continuous).

Incidence rate is per 1,000 person-years.

Abbreviation: CumMHR: cumulative monocyte to high-density lipoprotein cholesterol ratio; CumCRP: cumulative high-sensitivity C-reactivity protein; BMI: body mass index; FBG: fasting blood glucose; eGFR: estimated glomerular filtration rate; TG: triglyceride; LDL-C: low-density lipoprotein cholesterol.

# Table S9. Associations between joint exposure to CumMHR and CumCRP and incident diabetes stratified by hypertensive status in the exposure period

|  | **Combination of CumCRP and CumMHR，HRs (95% CIs)** | | | | | | ***P*-interaction** |
| --- | --- | --- | --- | --- | --- | --- | --- |
|  | **CumCRP<1 mg/L & CumMHR<0.2340** | **1≤ CumCRP <3 mg/L & CumMHR<0.2340** | **CumCRP≥3 mg/L & CumMHR<0.2340** | **CumCRP<1 mg/L & CumMHR≥0.2340** | **1≤CumCRP<3 mg/L & CumMHR≥0.2340** | **CumCRP≥3 mg/L & CumMHR≥0.2340** |  |
| **No-hypertension** | 1776/21080 |  |  |  |  | 0.0025 | 0.0021 |
| Event/Total | 220/4796 | 371/4458 | 174/1914 | 212/2951 | 461/4284 | 338/2677 |  |
| Incidence rate | 6.08 | 11.21 | 12.28 | 9.77 | 14.80 | 17.53 |  |
| Unadjusted model | Reference | 1.86(1.57–2.19) | 2.03 (1.67–2.48) | 1.60 (1.33–1.93) | 2.44 (2.08–2.86) | 2.90 (2.44–3.43) |  |
| Model 1 | Reference | 1.63(1.38–1.92) | 1.65(1.35–2.01) | 1.50(1.24–1.81) | 2.06(.74–2.42) | 2.22 (1.86–2.64) |  |
| Model 2 | Reference | 1.51(1.27–1.78) | 1.56(1.27–1.91) | 1.36(1.12–1.65) | 1.70(1.44–2.01) | 2.04 (1.70–2.45) |  |
| *P*-interaction: CumMHR (< median, or ≥ median) ×CumCRP (<1, 1 to 3, or ≥3 mg/L) = 0.0276; CumMHR (< median, or ≥ median) ×LogCumCRP<0.0001 | | | | | | |  |
| **Hypertension** | 3072/19733 |  |  |  |  |  |  |
| Event/Total | 307/2947 | 632/4106 | 326/2185 | 310/2226 | 790/4564 | 707/3705 |  |
| Incidence rate | 15.09 | 22.76 | 22.37 | 21.07 | 26.13 | 29.01 |  |
| Unadjusted model | Reference | 1.52(1.32–1.74) | 1.48(1.27–1.73) | 1.39 (1.18–1.62) | 1.72 (1.51–1.97) | 1.92 (1.68–2.19) |  |
| Model 1 | Reference | 1.38 (1.21–1.58) | 1.30(1.11–1.52) | 1.31 (1.11–1.53) | 1.49 (1.31–1.71) | 1.58 (1.38–1.81） |  |
| Model 2 | Reference | 1.34(1.16–1.53) | 1.30 (1.11–1.52) | 1.25(1.06–1.47) | 1.41 (1.22–1.61) | 1.50 (1.30–1.73) |  |
| *P*-interaction: CumMHR (< median, or ≥ median) ×CumCRP (<1, 1 to 3, or ≥3 mg/L)= 0.1724; CumMHR (< median, or ≥ median) ×LogCumCRP=0.0003 | | | | | | |  |

Model 1: adjusted for age (continuous), sex, education, smoking status, drinking status, physical activity, family history of diabetes, and BMI (categorical);

Model 2: Model 1+ logTG (continuous), LDL-C (continuous), eGFR (categorical), antihypertensives (yes or no), lipid-lowering drugs (yes or no), and log(leukocyte) (continuous).

Incidence rate is per 1,000 person-years.

Abbreviation: CumMHR: cumulative monocyte to high-density lipoprotein cholesterol ratio; CumCRP: cumulative high-sensitivity C-reactivity protein; BMI: body mass index; FBG: fasting blood glucose; eGFR: estimated glomerular filtration rate; TG: triglyceride; LDL-C: low-density lipoprotein cholesterol.

# Table S10. Associations between joint exposure to CumMHR and CumCRP with diabetes stratified by impaired fasting glucose status in the exposure period

|  | **Combination of CumCRP and CumMHR，HRs (95%CIs)** | | | | | | ***P*-interaction** |
| --- | --- | --- | --- | --- | --- | --- | --- |
|  | **CumCRP<1 mg/L & CumMHR<0.2340** | **1≤CumCRP<3 mg/L & CumMHR<0.2340** | **CumCRP≥3 mg/L & CumMHR<0.2340** | **CumCRP<1 mg/L & CumMHR≥0.2340** | **1≤CumCRP<3 mg/L & CumMHR≥0.2340** | **CumCRP≥3 mg/L & CumMHR≥0.2340** |  |
| **IFG (whichever FBG≥6.1 during exposure visits) (2166/7091)** | | | | | | | 0.0783 |
| Event/Total | 244/1148 | 456/1677 | 234/755 | 220/798 | 562/1597 | 450/1116 |  |
| Incidence rate | 32.67 | 44.66 | 52.86 | 45.31 | 59.94 | 73.15 |  |
| Unadjusted model | Reference | 1.36 (1.17–1.59) | 1.61 (1.35–1.93) | 1.37 (1.14–1.65) | 1.81 (1.56–2.11) | 2.22 (1.90– 2.59) |  |
| Model 1 | Reference | 1.27 (1.09–1.49) | 1.35 (1.12–1.61) | 1.40 (1.15–1.66) | 1.65 (1.41–1.92) | 1.75 (1.49– 2.05) |  |
| Model 2 | Reference | 1.23 (1.05–1.43) | 1.26 (1.05–1.51) | 1.25 (1.03–1.50) | 1.42 (1.21–1.69) | 1.43 (1.21–1.69) |  |
| *P*-interaction: CumMHR (< median, or ≥ median) ×CumCRP (<1, 1 to 3, or ≥3 mg/L) = 0.7347; CumMHR (< median, or ≥ median) ×LogCumCRP=0.0148. | | | | | | |  |
| **Non-IFG (2682/33722)** | | | | | | |  |
| Event/Total | 283/6595 | 547/6887 | 266/3344 | 302/4379 | 689/7251 | 595/5266 |  |
| Incidence rate | 5.77 | 10.80 | 10.94 | 9.57 | 13.25 | 15.87 |  |
| Unadjusted model | Reference | 1.89 (1.64–2.18) | 1.91 (1.61–2.26) | 1.65 (1.41–1.95) | 2.31(2.01–2.65) | 2.76 (2.40–3.18) |  |
| Model 1 | Reference | 1.64 (1.42–1.89) | 1.55 (1.30–1.83) | 1.52 (1.29–1.79) | 1.88 (1.63–2.16) | 2.07 (1.79–3.40) |  |
| Model 2 | Reference | 1.56 (1.35–1.80) | 1.53 (1.30–1.81) | 1.38 (1.17–1.63) | 1.64 (1.43–1.89) | 1.91 (1.64–2.22) |  |
| *P-*interaction: CumMHR (< median, or ≥ median) ×CumCRP (<1, 1 to 3, or ≥3 mg/L) = 0.0142; CumMHR (< median, or ≥ median) ×LogCumCRP<0.001 | | | | | | |  |

Model 1: adjusted for age (continuous), sex, education, smoking status, drinking status, physical activity, family history of diabetes, and BMI (categorical);

Model 2: Model 1+ hypertension (categorical), FBG (continuous), logTG (continuous), LDL-C(continuous), eGFR(categorical), antihypertensives (yes or no), lipid-lowering drugs (yes or no), and log(leukocyte)(continuous).

Incidence rate is per 1,000 person-years.

Abbreviation: CumMHR: cumulative monocyte to high-density lipoprotein cholesterol ratio; CumCRP: cumulative high-sensitivity C-reactivity protein; BMI: body mass index; FBG: fasting blood glucose; eGFR: estimated glomerular filtration rate; TG: triglyceride; LDL-C: low-density lipoprotein cholesterol.

# Table S11. Sensitivity analysis of associations between joint exposure to CumMHR and CumCRP and type 2 diabetes

|  | **Combination of CumCRP and CumMHR，HRs (95% CIs)** | | | | | |
| --- | --- | --- | --- | --- | --- | --- |
|  | **CumCRP<1 mg/L & CumMHR<0.2340** | **1≤CumCRP<3 mg/L & CumMHR<0.2340** | **CumCRP≥3 mg/L & CumMHR<0.2340** | **CumCRP<1 mg/L & CumMHR≥0.2340** | **1≤ CumCRP<3 mg/L & CumMHR≥0.2340** | **CumCRP≥3 mg/L & CumMHR≥0.2340** |
| **Exclude suspected infection** | 4285/36744 |  |  |  |  |  |
| Model 1 | Reference | 1.53 (1.37–1.70) | 1.51(1.31–1.73) | 1.43 (1.27–1.62) | 1.78(1.61–1.98) | 1.92(1.71–2.16) |
| Model 2 | Reference | 1.43 (1.29–1.59) | 1.39 (1.21–1.60) | 1.32 (1.16–1.49) | 1.54(1.38–1.71) | 1.72(1.52–1.95) |
| *P-*interaction: CumMHR (< median, or ≥ median) ×CumCRP (<1, 1 to 3, or ≥3 mg/L)= 0.0202; CumMHR (< median, or ≥ median) × LogCumCRP<0.001; | | | | | | |
| **Excluded baseline CVD** | 4498/38577 |  |  |  |  |  |
| Model 1 | Reference | 1.54 (1.38–1.72) | 1.51 (1.33–1.72) | 1.45(1.28– 1.64) | 1.83 (1.64–2.04) | 1.95 (1.74–2.18) |
| Model 2 | Reference | 1.45(1.30–1.62) | 1.45 (1.28–1.65) | 1.33(1.17–1.51) | 1.58 (1.41–1.76) | 1.77 (1.57–1.98) |
| *P*-interaction: CumMHR (< median, or ≥ median) ×CumCRP (<1, 1 to 3, or ≥3 mg/L) = 0.0340; CumMHR (< median, or ≥ median) ×LogCumCRP<0.001; | | | | | | |
| **Exclude events in first two years** | 3409/39374 |  |  |  |  |  |
| Model 1 | Reference | 1.52 (1.34–1.71) | 1.27 (1.10–1.48) | 1.47 (1.28–1.70) | 1.78 (1.58–2.01) | 1.70 (1.50–1.94) |
| Model 2 | Reference | 1.42 (1.25–1.60) | 1.24 (1.06–1.44) | 1.36 (1.18–1.57) | 1.55 (1.37–1.75) | 1.55 (1.36–1.77) |
| *P*-interaction: CumMHR (< median, or ≥ median) ×CumCRP (<1, 1 to 3, or ≥3 mg/L); CumMHR (< median, or ≥ median) ×LogCumCRP=0.0024 | | | | | | |
| **Excluded missing data** | 4778/40008 |  |  |  |  |  |
| Model 1 | Reference | 1.53 (1.36–1.72) | 1.46 (1.30–1.66) | 1.44 (1.28–1.63) | 1.79(1.60–2.03) | 1.90 (1.71–2.12) |
| Model 2 | Reference | 1.44(1.29–1.60) | 1.44 (1.27–1.63) | 1.35(1.19–1.52) | 1.59(1.43–1.76) | 1.75(1.56–1.96) |
| *P*-interaction: CumMHR (< median, or ≥ median) ×CumCRP (<1, 1 to 3, or ≥3 mg/L) = 0.0297; CumMHR (< median, or ≥ median) ×LogCumCRP=0.0587 | | | | | | |
| **Excluded statin use** | 4804/40583 |  |  |  |  |  |
| Model 1 | Reference | 1.53 (1.38–1.71) | 1.48 (1.31–1.68) | 1.43 (1.27–1.62) | 1.78 (1.61–1.98) | 1.89 (1.69–2.10) |
| Model 2 | Reference | 1.42 (1.28–1.60) | 1.43 (1.26–1.62) | 1.32 (1.17–1.50) | 1.55 (1.39–1.72) | 1.71 (1.53–1.92) |
| *P*-interaction: CumMHR (< median, or ≥ median) ×CumCRP (<1, 1 to 3, or ≥3 mg/L) = 0.0277; CumMHR (< median, or ≥ median) × LogCumCRP<0.0001 | | | | | | |

Model 1: adjusted for age (continuous), sex, education, smoking status, drinking status, physical activity, family history of diabetes, and BMI (categorical);

Model 2: Model 1 + FBG (continuous), hypertension (categorical), logTG (continuous), LDL-C (continuous), eGFR (categorical), antihypertensives (yes or no), lipid-lowering drugs (yes or no), *log*(leukocyte) (continuous).

Incidence rate is per 1,000 person-years.

Abbreviation: CumMHR: cumulative monocyte to high-density lipoprotein cholesterol ratio; CumCRP: cumulative high-sensitivity C-reactive protein; BMI: body mass index; FBG: fasting blood glucose; eGFR: estimated glomerular filtration rate; TG: triglyceride; LDL-C: low-density lipoprotein cholesterol.

# Table S12. Incidence of diabetes according to joint exposure to BasCRP and BasMHR

|  | **Combination of BasCRP and BasMHR，HR(95%)** | | | | | |
| --- | --- | --- | --- | --- | --- | --- |
|  | **BasCRP<1 mg/L & BasMHR<0.2162** | **1≤ BasCRP <3 mg/L & BasMHR<0.2162** | **BasCRP≥3 mg/L & BasMHR<0.2162** | **BasCRP<1 mg/L & BasMHR≥0.2162** | **1≤ BasCRP <3 mg/L & BasMHR≥0.2162** | **BasCRP≥3 mg/L & BasMHR≥0.2162** |
| DIABETES | 884/10474 | 736/6923 | 409/2996 | 1064/8805 | 910/6380 | 845/5235 |
| Incidence rate | 11.60 | 14.84 | 19.73 | 17.09 | 20.87 | 24.00 |
| Model(unadjusted) | Reference | 1.28 (1.16–1.41) | 1.69 (1.50–1.90) | 1.45 (1.32–1.58) | 1.80 (1.62–1.95) | 2.04 (1.86–2.25) |
| Model 1 | Reference | 1.10 (1.00–1.21) | 1.39 (1.23–1.57) | 1.33 (1.21–1.46) | 1.38 (1.25–1.51) | 1.53 (1.39–1.69) |
| Model 2 | Reference | 1.00 (0.91–1.10) | 1.21 (1.08–1.37) | 1.17 (1.06–1.29) | 1.15 (1.04–1.27) | 1.28 (1.15–1.42) |
| Model 3 | Reference | 0.95 (0.86–1.04) | 1.11 (0.99–1.26) | 1.18 (1.07–1.29) | 1.08 (0.97–11.9) | 1.15 (1.04–1.28) |
| *P*-interaction: BasMHR (< median, or ≥ median) × BasCRP (<1, 1 to 3, or ≥3 mg/L) = 0.3833; BasMHR (< median, or ≥ median) ×LogBasCRP=0.0027 | | | | | | |

Model 1: adjusted for age (continuous), sex, education, smoking status, drinking status, physical activity, family history of diabetes, and BMI (categorical);

Model 2: Model 1 + FBG (continuous), hypertension (categorical), logTG (continuous), LDL-C(continuous), eGFR(categorical), antihypertensives (yes or no), lipid-lowering drugs (yes or no), and log(leukocyte)(continuous).

Incidence rate is per 1,000 person-years.

Abbreviation: BasMHR: baseline monocyte to high-density lipoprotein cholesterol ratio; BasCRP: baseline high-sensitivity C-reactivity protein; BMI: body mass index; FBG: fasting blood glucose; eGFR: estimated glomerular filtration rate; TG: triglyceride; LDL-C: low-density lipoprotein cholesterol.

# Table S13 C-statistics for incident diabetes predicted by the relevant risk factors and addition of CumMHR in each CumCRP stratum

|  | **Event/Total** | **Models** | **C-statistics (95% CI)** | **SE** |
| --- | --- | --- | --- | --- |
| CumCRP<1 mg/L | 1049/12920 | Multivariable model | 0.7596 (0.7447–0.7746) | 0.00763 |
|  |  | Multivariable model + baseline MHR | 0.7616 (0.7467–0.7764) | 0.00757 |
|  |  | Multivariable model + cumulative MHR | 0.7621 (0.7473–0.7769) | 0.00756 |
| 1≤CumCRP<3 mg/L | 2254/17412 | Multivariable model | 0.7247 (0.7135–0.7358) | 0.00568 |
|  |  | Multivariable model + baseline MHR | 0.7248 (0.7136–0.7359) | 0.00569 |
|  |  | Multivariable model + cumulative MHR | 0.7255 (0.7143–0.7366) | 0.00568 |
| CumCRP>3 mg/L | 1545/10481 | Multivariable model | 0.7328 (0.7194–0.7461) | 0.00680 |
|  |  | Multivariable model + baseline MHR | 0.7328 (0.7194–0.7461) | 0.00680 |
|  |  | Multivariable model + cumulative MHR | 0.7345 (0.7212–0.7478) | 0.00680 |

The Multivariable model was adjusted for age (continuous), sex, education, smoking status, drinking status, physical activities, family history of diabetes, BMI (categorical), FBG (continuous), hypertension (categorical), *log*TG (continuous), LDL-C (continuous), eGFR (categorical), antihypertensives (yes or no), lipid-lowering drugs (yes or no), log(leukocyte) (continuous), and *log*(hsCRP).

Abbreviation as Table S3.
